# Cytochrome P450 diversity and induction by gorgonian allelochemicals in the marine gastropod *Cyphoma gibbosum*

**DOI:** 10.1186/1472-6785-10-24

**Published:** 2010-12-01

**Authors:** Kristen E Whalen, Victoria R Starczak, David R Nelson, Jared V Goldstone, Mark E Hahn

**Affiliations:** 1 Biology Department, Woods Hole Oceanographic Institution, Woods Hole, MA 02543, USA; 2 Department of Molecular Sciences, University of Tennessee, Memphis, TN 38163, USA; 3 Scripps Institution of Oceanography, University of California San Diego, La Jolla, CA 92093

## Abstract

**Background:**

Intense consumer pressure strongly affects the structural organization and function of marine ecosystems, while also having a profound effect on the phenotype of both predator and prey. Allelochemicals produced by prey often render their tissues unpalatable or toxic to a majority of potential consumers, yet some marine consumers have evolved resistance to host chemical defenses. A key challenge facing marine ecologists seeking to explain the vast differences in consumer tolerance of dietary allelochemicals is understanding the biochemical and molecular mechanisms underlying diet choice. The ability of marine consumers to tolerate toxin-laden prey may involve the cooperative action of biotransformation enzymes, including the inducible cytochrome P450s (CYPs), which have received little attention in marine invertebrates despite the importance of allelochemicals in their evolution.

**Results:**

Here, we investigated the diversity, transcriptional response, and enzymatic activity of CYPs possibly involved in allelochemical detoxification in the generalist gastropod *Cyphoma gibbosum*, which feeds exclusively on chemically defended gorgonians. Twelve new genes in CYP family 4 were identified from the digestive gland of *C. gibbosum*. Laboratory-based feeding studies demonstrated a 2.7- to 5.1-fold induction of *Cyphoma *CYP4BK and CYP4BL transcripts following dietary exposure to the gorgonian *Plexaura homomalla*, which contains high concentrations of anti-predatory prostaglandins. Phylogenetic analysis revealed that *C. gibbosum *CYP4BK and CYP4BL were most closely related to vertebrate CYP4A and CYP4F, which metabolize pathophysiologically important fatty acids, including prostaglandins. Experiments involving heterologous expression of selected allelochemically-responsive *C. gibbosum *CYP4s indicated a possible role of one or more CYP4BL forms in eicosanoid metabolism. Sequence analysis further demonstrated that *Cyphoma *CYP4BK/4BL and vertebrate CYP4A/4F forms share identical amino acid residues at key positions within fatty acid substrate recognition sites.

**Conclusions:**

These results demonstrate differential regulation of CYP transcripts in a marine consumer feeding on an allelochemical-rich diet, and significantly advance our understanding of both the adaptive molecular mechanisms that marine consumers use to cope with environmental chemical pressures and the evolutionary history of allelochemical-metabolizing enzymes in the CYP superfamily.

## Background

The dominance of gorgonian corals (Cnidaria, Octocorallia, Gorgonacea) on Caribbean reefs can be attributed, in part, to their chemical defenses, which limit predation by most potential consumers [1,2]. Among the classes of natural products isolated from gorgonians are acetogenins, prostanoids, highly functionalized steroids, and a diverse suite of terpenoid derivatives (e.g., diterpenes and sesquiterpenes) [3,4], all of which contain representatives with anti-predatory activity (reviewed in [5]). Despite the unique toxicological challenge that gorgonians present to marine consumers, the ovulid gastropod *Cyphoma gibbosum *thrives solely on a diet of allelochemically-rich octocorals [6], implying that this obligate consumer has evolved mechanisms of allelochemical tolerance. Additionally, *C. gibbosum*'s tissues do not mirror the chemical composition of its octocoral prey [7], suggesting that this generalist consumer can biotransform dietary secondary metabolites.

Elucidating the molecular mechanisms governing allelochemical resistance is crucial for understanding the genetic basis of adaptation in consumers like *C. gibbosum *that regularly feed on chemically defended prey. In marine systems, however, knowledge of genes conferring consumer tolerance to dietary allelochemicals is in its infancy [8] compared to that of terrestrial systems [9,10]. Metabolic resistance of insects to host plant toxins is known to involve a battery of cytochrome P450 (CYP) enzymes that oxidize a variety of hydrophobic compounds including dietary allelochemicals [11]. Much of the apparent diversity of CYP isoforms in insects is likely due to reciprocal evolutionary influences between host chemical defenses and consumer detoxification mechanisms, aptly termed a co-evolutionary "arms race" [12,13]. According to this view, multiple gene duplication and adaptive divergence of consumer CYP genes have allowed isoforms to gain new functions and increase the range of possible substrates while retaining ancestral metabolic capabilities [14]. For generalist consumers that must cope with a diversity of allelochemicals, having an assortment of catalytically versatile cytochrome P450 genes may provide a competitive advantage in dealing with the unpredictability of prey chemical defenses [15].

Consumer allelochemical tolerance is driven not only by the genetic and functional diversity of cytochromes P450, but also has been correlated with the elevated transcription of CYP genes [16]. In vertebrate systems, CYP induction following exposure to dietary xenobiotics (including allelochemicals) primarily involves genes within CYP families 1 through 4 [17], while in insects allelochemically inducible forms are found chiefly in CYP families 4, 6 and 9 [11]. The identification of molluscan CYP genes involved in allelochemical metabolism has proved difficult because many of the CYP families previously identified in such reactions are either taxon-specific (i.e., insect-specific CYP 6 and 9) [18], or lack full-length sequenced representatives from protostomes in general, including molluscs (i.e., CYP1, 2 and 3) [19,20]. In contrast, members of the CYP4 family represent a substantial portion of the CYP cDNA sequences identified to date in both arthropods and molluscs [18,21,22].

The CYP4 family is considered one of the most ancient CYP families, having evolved from the steroid-synthesizing CYPs and since diverged into an array of subfamilies and genes encoding enzymes acting on diverse substrates [23]. In vertebrates, CYP4 genes are the predominant fatty acid ω-hydroxylases, preventing lipotoxicity by hydroxylating eicosanoids, including prostaglandins [24]. Prostaglandins are potent signaling molecules, known as regulators of fever, inflammation, and pain response in human biology. In marine systems, prostaglandins function as feeding deterrents and can comprise up to 8% of the wet weight of some gorgonian species (reviewed by [25]). Yet the diversity of CYP4 substrates extends far beyond fatty acid derivatives to include anti-herbivory isoquinoline alkaloids [26] and terpene derivatives, including sesquiterpenoids [27]. The capacity of CYP4 genes to metabolize terpenoid derivatives is of particular interest due to the predominance of defensive diterpenoid and sesquiterpenoid compounds across all members of the Octocorallia [28]. This overlap between the diverse pool of CYP4 substrates and the major structural classes of gorgonian natural products make the CYP4 family an appropriate target for further investigation with respect to allelochemical detoxification in molluscan consumers.

An initial investigation by Vrolijk and Targett [29] was the first to suggest that the ability of *C. gibbosum *to tolerate dietary allelochemicals may involve enhanced biotransformation by enzymes such as cytochromes P450. The authors measured both P450 content and activity from field-collected *C. gibbosum *feeding on four species of gorgonian corals (*Briareum asbestinum, Gorgonian ventalina, Plexaura homomalla*, and *Pseudopterogorgia americana*). However, P450-specific content in *C. gibbosum *digestive gland was low and only quantifiable in individuals collected from *P. americana*. Moreover, two spectrofluorometric assays (methoxyresorufin O-deethylase (MROD) and ethoxyresorufin O-deethylase (EROD)), classically used to detect CYP1A-specific activity in vertebrates exposed to anthropogenic pollutants, failed to detect the presence of CYP activity in *C. gibbosum *digestive gland. Since publication of that paper, advances in the field of molecular ecology warrant a re-evaluation of this hypothesis. Using a targeted molecular approach, we identified putative allelochemical-responsive *C. gibbosum *CYP4 cDNAs, measured their transcript expression following allelochemical exposure, and assessed their ability to metabolize diagnostic substrates. Evidence presented here emphasizes the role of CYP genes in the adaptation of marine consumers to their chemically defended prey.

## Methods

### Animal collection and feeding assay design

**Figure 1:**
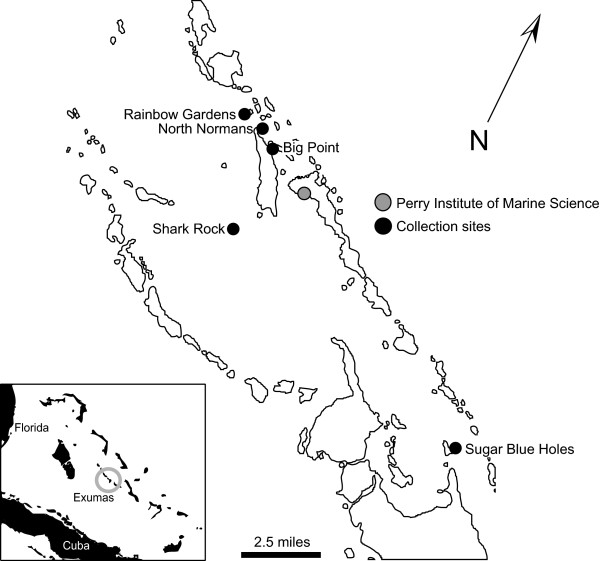
***Cyphoma *and gorgonian sampling locations in the Exuma Keys, Bahamas**. Black circles indicate the location of reefs where animals were collected.

A total of 151 adult *Cyphoma gibbosum *(ca 2-3 cm length) were collected from five shallow reefs (< 20 m) (Big Point - 23°47.383'N, 76°8.113'W; North Normans - 23°47.383'N, 76°8.264'W; Rainbow Gardens - 23°47.792'N, 76°8.787'W; Shark Rock - 23°45.075'N, 76°7.475'W; Sugar Blue Holes - 23°41.910'N, 76°0.23'W) surrounding the Perry Institute of Marine Science (PIMS), Lee Stocking Island, Exuma Cays, Bahamas (Figure 1) in January 2006. Snails were immediately transported to wet laboratory facilities provided by PIMS where a series of feeding assays were conducted with seven gorgonian species (*Briareum asbestinum, Eunicea mammosa, Gorgonia ventalina, Pseudopterogorgia acerosa, Pseudopterogorgia americana, Pseudopterogorgia elisabethae, Plexaura homomalla*) observed to serve as hosts for *C. gibbosum *in the field.

**Figure 2:**
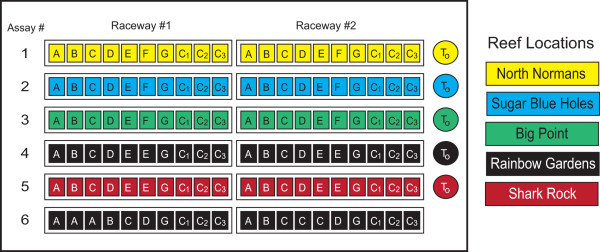
**Feeding assay experimental design for snails collected for RNA isolation**. Snails were randomly placed into one of nine groups, time zero group (T_o_), a control group (C_1_,C_2_, C_3_), or one of seven gorgonian diet groups (A, *Briareum asbestinum*; B, *Eunicea mammosa*; C, *Gorgonia ventalina*; D, *Pseudopterogoria acerosa*; E, *Pseudopterogoria americana*; F, *Pseudopterogoria elisabethae*; G, *Plexaura homomalla*). Digestive glands collected after completion of this feeding assay were used for RNA isolation and mRNA expression analysis.

Individual snails were housed separately in 3-L polycarbonate tanks which were placed in a 12' × 20" raceway supplied with filtered, continuous-flow seawater at a flow rate of approximately 1 L min^-1^. This design allowed for a common water source to feed each tank but prevented mixing between tanks. Snails collected from the same reefs were housed separately in the same raceways and randomly assigned to one of nine groups -- one of seven gorgonian diets, a control diet, or a time-zero group -- at the start of the feeding assays (Figure 2). Snails assigned to the time zero groups were dissected within two hours after field collection and digestive glands were preserved in RNA Later^® ^(Ambion, Austin, TX) and stored at -80°C. Time-zero snails provide baseline information about the CYPs expressed in a population of *C. gibbosum *on a particular reef at the time of collection. The remaining snails were maintained on their respective diet (gorgonian or control) for a total of four days, reflecting the mean residence time of *C. gibbosum *on gorgonian colonies [30].

Gorgonian colonies were collected from shallow reefs (< 20 m) surrounding PIMS and housed in a separate raceway prior to introduction into the tanks containing *C. gibbosum*. To ensure representation of gorgonian chemotypes, if they exist, a minimum of ten colonies were used in the feeding assays. Gorgonian colonies were cut into 2-3 inch pieces and introduced into the feeding assays less than 12 hours after field collection. The control diet consisted of alginic acid and freeze-dried squid powder prepared as described in [2]; this diet mirrored the average nutritional quality and consistency of gorgonian tissue. The squid-alginate paste was pressed into sixteen 3-mm deep wells drilled into a 3" × 1" piece of Formica^® ^resembling a domino. The domino was then placed into a 0.25 M calcium chloride solution allowing the squid-alginate paste to harden. Both control and gorgonian diets were replaced every 24 hours for four days, and feeding activity was monitored by the presence of feeding scars on their gorgonian prey and empty wells on control dominos (see Additional file 1 for a summary of digestive glands collected). Upon completion of the 4 day feeding assay, the digestive glands were immediately dissected, weighed, preserved in RNA Later^® ^and maintained at -80°C until further analysis. A second set of feeding assays running in parallel to those described above are detailed in [31]. Material obtained from this subsequent assay was used to investigate the enzymatic activity of *C. gibbosum *digestive gland microsomes.

### Initial cDNA cloning

In 2004, a preliminary series of feeding assays at PIMS with 15 adult *C. gibbosum *feeding on four gorgonian species (*B. asbestinum, G. ventalina, P. acerosa, P. americana*) provided the material for the initial cloning of CYP4 cDNA fragments. RNA isolation, RT-PCR with degenerate primers, cDNA cloning, and sequencing are described in detail in Additional file 2.

### Rapid amplification of cDNA ends (RACE)

Gene specific primers designed to the three initial cDNA fragments (see Additional file 3) were used in combination with adaptor-specific primers to obtain full-length CYP sequences by 5'- and 3'-RACE-PCR. Digestive gland total RNA from seven snails collected during the January 2006 feeding assays was purified and DNAse treated using the RNeasy Maxi Kit and RNase-free DNAse Kit (Qiagen, Valencia, CA) following the manufacturer's instructions. Poly(A)+ RNA was isolated using the MicroPoly(A) Purist mRNA purification kit according to the manufacturer's instructions and equal amounts of poly(A)+ RNA from each of the seven snails feeding either on a control diet or one of six gorgonian species (0.14 μg poly(A)+ RNA/individual) was pooled to ensure representation of all CYPs expressed under various dietary conditions. One microgram of pooled poly(A)+RNA was primed with modified oligo (dT) primers and used to create an adaptor-ligated double-stranded cDNA library synthesized using the Marathon cDNA Amplification Kit (BD Biosciences, Palo Alto, CA) according to the manufacturer's instructions. Amplification of PCR products was carried out according to the Advantage 2 PCR Enzyme Kit (Clontech, Mountain View, CA) and cycling parameters were as follows: 94°C for 30 sec; 5 cycles of 94°C for 5 sec, 72°C for 2.5 min; 5 cycles of 94°C for 5 sec, 70°C for 2.5 min; 25 cycles of 94°C for 5 sec, 68°C for 2.5 min; 68°C for 5 min with the following specific primer pairs (RACE_1_F/CYP4_A12_R; RACE_1_F/CYP4_D09_F; RACE_1_F/CYP4_F11_R; RACE_1_F/CYP4_F1; and AP1/CYP4-3_R1). Once the initiation and termination codons had been identified, primers were designed to amplify the full-length CYP4 cDNAs using PfuUltra™ Fusion HS DNA Polymerase (Stratagene, La Jolla, CA) with the following cycling parameters: 95°C for 1 min; 40 cycles of 95°C for 20 sec, 63°C for 20 sec, 72°C for 1 min; 72°C for 3 min with specific primers pairs (CYP4-3_F3/CYP4-3_R6, and CYP4-2_F1/CYP4-2_R1). All PCR products were clones and sequenced as described in Additional file 2.

### Sequence alignments and phylogenetic analysis

Partial and full-length CYP4 nucleotide sequences were clustered by Sequencher 4.6 based on nucleotide identity (> 80% identity), aligned using ClustalX [32] and this alignment was used to construct maximum parsimony trees using PAUP*4.0b10 [33]. The number of possible distinct CYP4 loci was inferred from tree topologies and pairwise comparisons of cDNA sequences. Sequences within each cluster were grouped according to maximum parsimony tree results, and consensus nucleotide and deduced amino acid sequences were generated from these groupings in BioEdit v7.0.5.2 [34]. Multiple alignments of *Cyphoma *CYP4 deduced amino acid sequences and other full-length metazoan CYP4 protein sequences were performed by ClustalX for phylogenetic analysis by Bayesian and maximum likelihood methods. A Bayesian phylogeny was generated using MrBayes (v 3.1.2; [35] with two independent runs of 3×10^6 ^generations each (sampled every 100^th ^generation) and a burn-in of 1×10^6 ^generations. Monte Carlo Markov chains estimates with uninformative prior probabilities were performed using the WAG model of amino acid substitution [36] and prior uniform gamma distributions approximated with four categories (WAG+I+gamma). Maximum likelihood phylogenetic relationships were calculated with the Pthreads version of RAxML v7.0.0 [37,38] using the WAG+gamma model of amino acid substitution. Multiple initial ML searches were performed from random starting points and bootstrap support was estimated for the best ML tree. Trees were visualized and manipulated using FigTree v1.1[39].

### Homology modeling

Amino acid sequences of *C. gibbosum *CYP4BK1 and CYP4BL3 were aligned to human CYP3A4 (PDB ID 1TQN) for which crystal structures are available [40]. Human CYP3A4 was selected as the template for constructing models because the two *Cyphoma *CYP4 proteins share the highest degree of amino acid sequence identity (23.9% for CYP4BK1, 24.4% for CYP4BL3) with CYP3A4. Homology models were constructed using Modeller (v 9.2, r5542; [41]), from alignments generated using ClustalW [32] and refined using salign_2d (Modeller). Default parameters in Modeller were applied, excluding water molecules and any ions that were part of any of the templates with the exception of the heme and heme iron. Homology model quality was assessed with DOPE (Modeller) and PROCHECK [42]. Evaluation of potential substrate access channels was performed using CAVER [43], while residues within the active sites were defined based on cavity detection using CASTp [44]. Putative active site residues were assessed based on a simple physical proximity metric of ≤ 5 Å from the oxo-heme moiety.

### Real-time quantitative RT-PCR

CYP4 transcript expression levels in 141 *C. gibbosum *digestive glands were quantified by real-time quantitative PCR using the iCycler MyiQ Real-Time PCR Detection System (Bio-Rad). Digestive gland total RNA from 141 individual snails participating in the 2006 feeding assays was purified and DNAse treated using the RNeasy Maxi Kit and RNase-free DNAse Kit (Qiagen) following the manufacturer's instructions. Poly(A)+ RNA was isolated using the MicroPoly(A) Purist mRNA purification kit according to the manufacturer's instructions. DNAse-treated poly(A)+ RNA (0.2 μg) was used to synthesize cDNA as described by the iScript™ cDNA Synthesis Kit (Bio-Rad, Hercules, CA). Quantitative RT-PCR reactions contained 12.5 μL 2× SYBR Green Supermix reagent (Bio-Rad), 10 ng cDNA, and 100 nM of each gene-specific primer in a final volume of 25 μL. A list of sequence-specific primers for quantitative PCR analysis of CYP4 transcript expression, a depiction of the transcripts recognized by primers targeted to a subset of CYP4BL cDNA sequences, and additional details on qRT-PCR conditions and statistical analysis can be found in Additional files 4, 5 and 6.

### Heterologous expression

Five *C. gibbosum *CYP4 sequences (CYP4BK1, CYP4BK2, CYP4BL1, CYP4BL3, and CYP4BL4) were expressed using a yeast heterologous expression system (see Additional file 7). Full length CYP4 sequences were amplified with custom primers (see Additional file 8) and cloned into the entry vector pENTR/D/TOPO (Invitrogen, Carlsbad, CA) then transferred to the expression vector pYESDEST52/V5-His using the TOPO cloning Kit (Invitrogen), and transformed into the *Saccharomyces cerevisiae *W(R) strain, which over-expresses the yeast NADPH-cytochrome P450 reductase gene under the control of a galactose-inducible promoter [45]. The W(R) yeast was made competent using the *S.c*. EasyComp™ transformation kit according to the manufacturer's instructions (Invitrogen), and separately transformed with each of the five *C. gibbosum *CYP expression clones and the control *Arabidopsis *β-glucuronidase (gus) gene. Yeast cells were grown to high density with glucose as the main carbon source, after which galactose was added to induce CYP expression as described in [45]. Yeast cells were harvested from 500 mL cultures after 8 and 15 hrs on inductive media, pelleted and used in the preparation of microsomes.

Yeast cells were suspended in degassed TES50 buffer (50 mM Tris-HCl, 1 mM EDTA, 0.6 M sorbitol; pH 7.4) containing 1.0 mM dithiothreitol (DTT) and protease inhibitor cocktail (1X) (Sigma) and mechanically disrupted using a BeadBeater (BioSpec Products, Inc., Bartlesville, OK) as described in [46]. The cell lysate was separated from the glass beads by decantation and centrifuged at 750 × g for 10 min then, without stopping, at 12,000 × g for 10 min using a Beckman J2-21 centrifuge (Fullerton, CA). The resulting microsomal pellet was resuspended in 1 to 2 mL of TEG50 buffer (50 mM Tris-HCl, 1 mM EDTA, 20% glycerol (by vol.), 1 mM DTT; pH 7.4) by gentle hand homogenization using a Potter-Elvehjem homogenizer and microsomal suspensions were stored at -80°C until use. The Bradford assay was used to determine microsomal protein concentration [47].

### Preparation of digestive gland microsomes

Individual *C. gibbosum *digestive glands were homogenized 1:4 (w/v) in ice-cold homogenization buffer (0.1 M potassium phosphate, 1 mM EDTA, 1 mM DTT, 1.15% potassium chloride, protease inhibitor cocktail (1X); pH 7.5) with an IKA Ultra Turrax T8 homogenizer (Wilmington, NC) for 30 sec on ice. All subsequent steps were carried out at 4°C. Cytosol was isolated by centrifugation of the crude homogenate at 750 × *g *for 10 min then, without stopping, at 12,000 × *g *for 10 min using a Beckman J2-21 centrifuge (Fullerton, CA). The supernatant was then transferred to an ultracentrifuge tube and centrifuged at 100,000 × *g *for 70 min using a Beckman L8-60M ultracentrifuge (Fullerton, CA). The microsomal pellet was resuspended in 0.2 to 0.8 mL of microsomal buffer (0.1 M potassium phosphate, 1 mM EDTA, 1 mM DTT, 20% glycerol (by vol.), pH 7.5) and stored at -80°C until use. An aliquot of suspension was taken for microsomal protein determination using the Bradford assay method [47] with BSA as the standard. Only those microsomal fractions from snails feeding on the control diet (n = 10) and *P. homomalla *(n = 4) were analyzed in this study.

### Fatty acid metabolism

Lauric acid hydroxylase activity was determined by the method described in [48]. The reaction mixture (0.1 mL final volume) contained 20 μL yeast microsomal protein (1.1-4.0 mg/mL) expressing *C. gibbosum *CYP4s, 1.3 mM NADP+, 3.3 mM glucose-6-phosphate, 0.4 U/mL glucose-6-phosphate dehydrogenase, 3.3 mM MgCl_2_, 0.1 mM [^14^C]-lauric acid in 100 mM Tris buffer (pH 7.5). Human liver microsomes (BD Biosciences) (1.0 mg/mL) were used as a positive control. Lauric acid and the ω-hydroxylated metabolite were detected by liquid scintillation counting.

Leukotriene B_4 _hydroxylase activity was determined by monitoring the conversion of LTB_4 _at absorbance 270 nm to its hydroxylated metabolites by recombinant CYP4 proteins or *Cyphoma *microsomes. The reaction mixture (0.1 mL final volume) contained 0.2-1.0 mg/mL of snail microsomes or 2.0 mg/mL of yeast microsomes with 1.3 mM NADP+, 3.3 mM glucose-6-phosphate, 0.4 U/mL glucose-6-phosphate dehydrogenase, 3.3 mM magnesium chloride, 29.7 μM LTB_4 _in 100 mM KPO_4_. Human liver microsomes (0.5 mg/mL) were used as a positive control. Both the lauric acid and LTB_4 _hydroxylase assays were performed by BD Biosciences Discovery Labware Division, Woburn, MA. Additional details on both fatty acid metabolism assays can be found in Additional file 9.

P450 specific content and reductase activity was measured in both yeast microsomes expressing *Cyphoma *CYP4 proteins and digestive gland microsomes as described in Additional file 9.

## Results

### Identification and phylogenetic analysis of CYP4 genes

**Figure 3:**
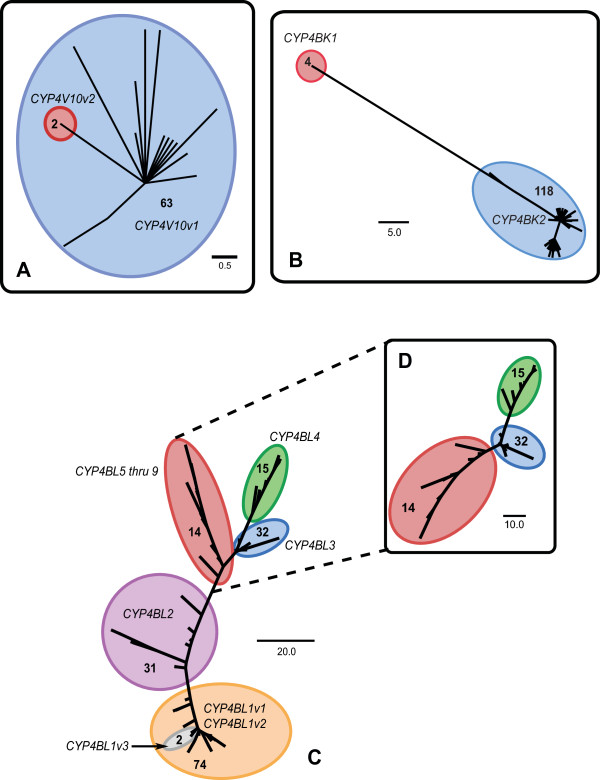
**Cluster analysis of clones within the CYP4V10, CYP4BK and CYP4BL subfamilies visualized by maximum parsimony**. Circles indicate putative genes or alleles within the CYP4V10 **(A)**, CYP4BK **(B) **and CYP4BL **(C, D) **subfamilies. Numbers within the circles indicate the number of clones representing each cluster. Scale bar indicates the number of nucleotide base changes.

An RT-PCR based cloning approach in combination with RACE was initially used to define the range of CYP4 genes expressed in *C. gibbosum*. This search identified three partial, putative CYP4 cDNAs of approximately 396 base pairs (bp) in length that shared 41-54% amino acid identity and were separated into three clusters designated CYP4-1, CYP4-2, and CYP4-3. Specific primers designed to each of the three CYP4 clusters were then used in 5' and 3' RACE reactions to obtain full-length sequences. In total, RACE and RT-PCR cloning efforts generated 352 cDNA clones distributed among the three clusters. Sequences within each cluster were aligned using ClustalX and used to construct three maximum parsimony trees to assist in the identification of possible distinct CYP4 genes (Figure 3).

**Figure 4:**
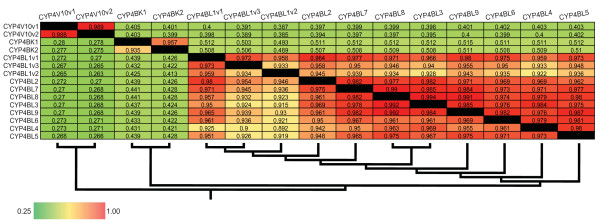
**Heat-map depicting percent identity between *Cyphoma *CYP4 cDNAs**. Values are shaded according to their percent identity with red and green indicating the highest and lowest degree of identity, respectively. Nucleotide percent identity is shown in the upper triangle while amino acid percent identity is shown in the lower triangle.

ClustalX alignments indicated that the *Cyphoma *CYP4 protein sequences share amino acid identities ranging from 26.3 to 99.2% (Figure 4). Phylogenetic analysis revealed that all of the *Cyphoma *sequences identified here belong to cytochrome P450 clan 4 and should be placed within the CYP4 family. From the analyses of tree topologies and pairwise comparisons of cDNA sequences we inferred the existence of fifteen full-length CYP4 cDNAs encoded by twelve distinct CYP4 loci. The individual cDNA sequences, designated CYP4V10v1, CYP4V10v2, CYP4BK1, CYP4BK2, CYP4BL1v1, CYP4BL1v2, CYP4BL1v3, CYP4BL2, CYP4BL3, CYP4BL4, CYP4BL5, CYP4BL6, CYP4BL7, CYP4BL8, and CYP4BL9 by the P450 nomenclature committee, have been deposited with [GenBank accession numbers EU546250 through EU546264]. The *Cyphoma *CYP4BK and CYP4BL sequences share less than 40% amino acid sequence identity with full-length vertebrate CYP4 members, but are included in the CYP4 family, in recognition of the ancient origin of the CYP4 family and the view that phylogenetic relationships need to be considered in assigning nomenclature [49]. Nine distinct CYP4BL genes (one with 3 allelic variants) were inferred in these analyses, and some of these share greater than 97% nucleotide sequence identity--the conventional cutoff for considering two sequences to be alleles at one locus [50]. Pairwise comparisons revealed patterns suggestive of concerted evolution by gene conversion, providing one possible explanation for the high sequence identity. However, we cannot rule out the possibility that there may be fewer loci than indicated here (see Additional file 10).

**Figure 5:**
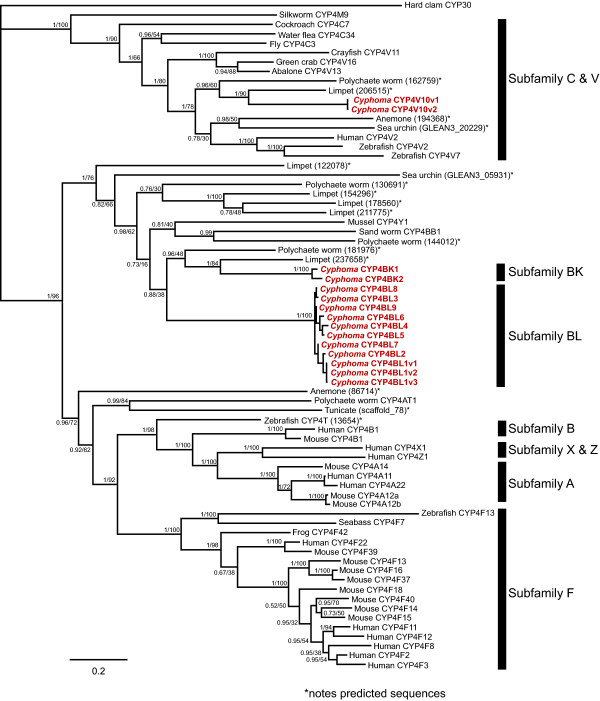
**Bayesian tree detailing relationships among *Cyphoma *and other invertebrate/vertebrate CYP4s**. Both Bayesian and Maximum Likelihood analyses resulted in trees with the same topology, therefore only the Bayesian tree is shown. Values at branch points represent posterior probabilities derived from 3×10^6 ^generations and maximum likelihood bootstrap values calculated with 1000 replications. Accession numbers are listed in Additional file 18.

Phylogenetic analysis also showed that the paralogous sequences within the CYP4BK and CYP4BL subfamilies fall within a larger clade containing CYP4 sequences from other molluscs, annelids, and an echinoderm, whose most closely related vertebrate homologs are found within CYP4 subfamilies A, B, F, T, X, and Z (Figure 5 and Additional file 11). Top scores from BlastX searches indicate that *Cyphoma *CYP4BK and CYP4BL sequences are most similar to mammalian CYP4F sequences rather than non-mammalian CYP4F members or other vertebrate CYP4 subfamilies. These findings suggest the possible convergent evolution between *Cyphoma *CYP4s and mammalian CYP4F forms, or, more likely, the retention in CYP4BK and CYP4BL of specific ancestral residues, which could indicate the possibility of shared substrates between these two groups.

### Quantitative RT-PCR analysis of CYP4 expression

Quantitative RT-PCR analysis was used to investigate the constitutive and inducible expression of CYP4 transcripts in *Cyphoma *after dietary exposure to several gorgonian species with varying allelochemical profiles (n = 6 to 33 snails examined per diet; 141 snails in total). Expression of CYP4 transcripts in digestive gland tissues was measured using general-cyp4V, general-cyp4BK, and general-cyp4BL primers. Attempts were made to design gene-specific primers for individual CYP4BL sequences; however, because of the high degree of sequence similarity this was not possible. Instead, two additional primer pairs specific for a subset of CYP4BL sequences, designated CYP4BL_(sub A) _and CYP4BL_(sub B) _were developed. Primer set CYP4BL_(sub A) _recognizes clones within the CYP4BL2, CYP4BL3 and CYP4BL4 clusters, while CYP4BL_(sub B) _primers pairs primarily recognize clones found in the CYP4BL1 and CYP4BL5 thru CYP4BL9 clusters (see Additional file 6). All transcript expression data were log transformed prior to statistical analysis to homogenize variances.

**Figure 6:**
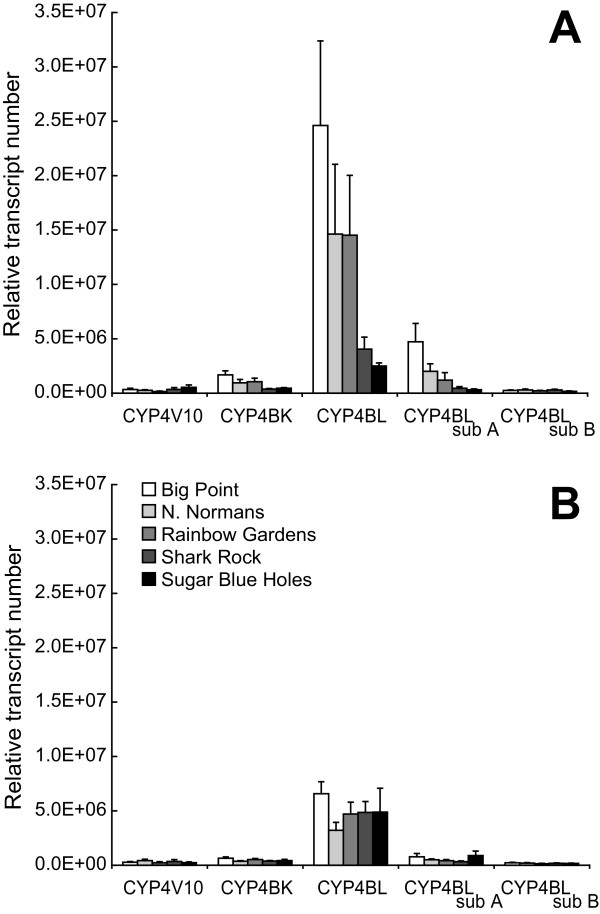
**CYP4 transcript expression is highly variable among time-zero snails in contrast to control-fed snails**. Bars represent the relative CYP4 transcript expression (mean ± SE) in **(A) **time-zero group snails (n = 31 total; Big Point (n = 6), North Normans (n = 6), Rainbow Gardens (n = 6), Shark Rock (n = 6) and Sugar Blue Holes (n = 7)), and **(B) **control snails (n = 33 total; Big Point (n = 6), North Normans (n = 6), Rainbow Gardens (n = 9), Shark Rock (n = 6), and Sugar Blue Holes (n = 6)). CYP4 transcript expression significantly differed among time-zero group snails collected from different reefs (*P *= 0.047, MANOVA), but did not vary among snails from different reefs fed a control diet for four days (*P *= 0.164, MANOVA).

Significant differences in CYP4 transcript expression were identified in snails in the time-zero group collected from different reefs (MANOVA, *P *< 0.05; Figure 6a and Additional file 12). In contrast, CYP4 transcript expression did not vary among snails collected from different reefs and fed a control diet for four days (MANOVA, *P *= 0.164, Figure 6b and Additional file 12). These results demonstrate that gene expression is highly variable among snails collected from different reefs, possibility reflecting the variability of gorgonian diets at each reef location. If one considers the time-zero group as a proxy for the natural variation of gene expression in individuals on different reefs, then four days feeding on a control diet is sufficient to allow gene expression to return to some basal level, no matter the reef of origin, once the allelochemical stimulus is removed. Therefore, these results support the use of control-fed snails as the true baseline, representing the non-allelochemically induced state of CYP4 gene expression in *Cyphoma*.

**Figure 7:**
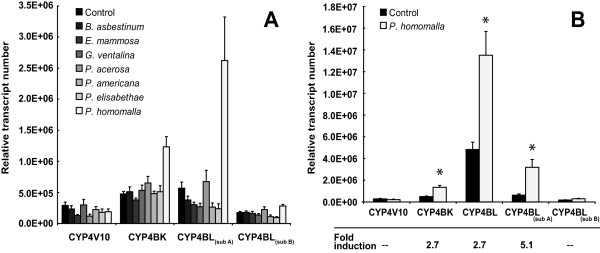
**Induction of CYP4 transcript expression in *Cyphoma *feeding on gorgonian diets**. **(A) **Bars represent the relative CYP4 gene expression (mean ± SE) in the digestive glands of snails collected from five reefs (Big Point, North Normans, Rainbow Gardens, Shark Rock and Sugar Blue Holes) and allowed to feed on *B. asbestinum *(n = 13 snails), *E. mammosa *(n = 12), *G. ventalina *(n = 13), *P. acerosa *(n = 10), *P. americana *(n = 12), *P. elisabethae *(n = 6), *P. homomalla *(n = 11) and the control diet (n = 33). **(B) **Bars represent the relative CYP4 transcript expression (mean ± SE) in the digestive glands of snails collected from four reefs (Big Point, North Normans, Rainbow Gardens and Sugar Blue Holes) and allowed to feed on a control diet (n = 27 snails) or *P. homomalla *(n = 9). CYP4BK, CYP4BL and CYP4BL_(sub A) _transcript expression was significantly higher in snails consuming *P. homomalla *(*P *< 0.001, ANOVA) than in control-fed snails.

Significant differences in CYP4 transcript expression were detected in snails feeding on *G. ventalina, P. americana *and *P. homomalla *compared to those feeding on control diets (MANOVAs, *P *< 0.001, see Figure 7a and Additional file 13). However, the differences in CYP4 expression in *P. americana*-fed snails were found not to be significant in ANOVA comparisons after P-values were adjusted using the Bonferrroni correction (see Additional file 14). For the remaining two gorgonian diets, *G. ventalina *and *P. homomalla*, a significant Diet × Reef interaction was observed, indicating that CYP4 expression in snails in response to the treatment diet was strongly influenced by the reef from which snails were obtained (for all MANOVAs, *P *< 0.001; see Additional file 13). ANOVA analysis further revealed that the significant Diet × Reef interaction could be traced to differences in CYP4BL_(sub A) _transcript expression among reefs. Two snails collected from N. Normans out of thirteen feeding on *G. ventalina*, and one snail collected from Shark Rock out of eleven feeding on *P. homomalla*, showed negligible expression of CYP4BL_(sub A) _transcripts, accounting for the significance of the interaction term. However, all three of these snails did express transcripts detected by subset-specific CYP4BL_(sub B) _primers at levels comparable to other snails feeding on the same diet, indicating that other CYP4BL subfamily transcripts were being expressed. Furthermore, when CYP4BL transcript levels were measured with general-cyp4BL primers in the single anomalous snail from Shark Rock feeding on *P. homomalla*, expression of CYP4BL transcripts was comparable to other individuals feeding on *P. homomalla*, and was higher than values obtained from snails feeding on diet controls. This finding indicates that while overall CYP4BL expression is elevated in *P. homomalla*-fed snails, certain genes within the CYP4BL subfamily are selectively induced.

In light of this information, CYP4BL transcript expression was measured in all *G. ventalina*, *P. homomalla *and control-fed snails with general-cyp4BL primers, and the data were reanalyzed excluding Shark Rock reef, which contained snails with negligible CYP4BL_(sub A) _transcript expression levels. There were no significant differences in CYP4 transcript expression between snails feeding on control and *G. ventalina *diets (data not shown). However, CYP4 transcript expression was still significantly induced in snails feeding on *P. homomalla*: CYP4BK, CYP4BL and CYP4BL_(sub A) _transcript expression levels were 2.7--5.1-fold greater in *C. gibbosum *feeding on *P. homomalla *as compared to controls (MANOVA/ANOVA, *P *< 0.001, 7b and Additional files 13 and 14).

### Results of enzymatic assays

To further investigate the possible link between gene induction and enhanced enzymatic activity, we examined CYP4-associated fatty acid monooxygenase activities. Digestive gland microsomes from snails consuming a diet of *P. homomalla *had significantly higher LTB_4 _hydroxylase activity in comparison to snails feeding on control diets. Nine out of ten snails feeding on the control diet displayed no measureable LTB_4 _hydroxylase activity, while three of the four snails feeding on *P. homomalla *recorded activity averaging 0.63 ± 0.47 pmol mg^-1 ^min^-1 ^(mean ± SE) (see Additional file 15). To identify the CYP isoforms responsible for this activity, we performed enzymatic analysis of recombinant yeast microsomes expressing CYP4BK1, CYP4BK2, CYP4BL1, CYP4BL3 or CYP4BL4. LTB_4 _hydoxylase activity was present in yeast expressing CYP4BL1 and CYP4BL3 with values ranging from 0.017 - 0.299 pmol mg^-1^min^-1 ^(see Additional file 16). The results of the enzymatic analysis in combination with the transcript data indicate that one or more CYP4BL isoforms that are inducible by *P. homomalla *possess eicosanoid hydroxylase activity.

### Analysis of substrate access channels and active site residues

To obtain insight into the structural features of *Cyphoma *CYP4 proteins that may determine their substrate specificities, we performed homology modeling of CYP4BL3 and CYP4BK1 and conducted a detailed analysis of multiple alignments of all *Cyphoma *CYP4 proteins. The homology models revealed differences in the substrate access channels and catalytic sites of CYP4BL3 and CYP4BK1 (see Additional file 17). The amino acid sequence alignment indicated that 55% of the sequence variation within the CYP4BK and CYP4BL subfamilies falls within or near the substrate recognition sites (SRSs) critical for defining the range of substrates metabolized by CYPs [51] (see Additional file 18), suggesting diversification of substrate specificities among closely related *Cyphoma *CYP4 proteins. Specific residues that have been shown to be important for hydroxylation of prostaglandins and fatty acids are conserved in the *Cyphoma *CYP4 genes; a detailed description of these can be found in Additional file 10.

## Discussion

This study provides the first evidence linking the induction of specific CYP transcript expression and corresponding enzymatic activity in a marine consumer to differences in gorgonian prey and, by inference, to specific differences in gorgonian chemical profiles. The diet of the generalist *C. gibbosum *includes a variety of gorgonian families with structurally diverse allelochemical profiles. Only snails feeding on the gorgonian *Plexaura homomalla *displayed both a significant induction (2.7- to 5.1-fold) of CYP4BK and CYP4BL transcripts and a corresponding increase in the metabolism of the diagnostic eicosanoid LTB_4 _in comparison to individuals feeding on a control diet devoid of gorgonian allelochemicals. Our results are consistent with the possibility that allelochemicals in *P. homomalla *induce *Cyphoma *CYP4 enzymes that may serve to detoxify the gorgonian allelochemicals.

*Plexaura homomalla *tissues contain high concentrations of prostaglandins (up to 8% of the wet tissue weight), which are unique to this genus of Caribbean soft coral [52-54]. In mammalian systems, prostaglandins behave as 'local' (i.e., autocrine or paracrine) lipid mediators, acting through G-protein-coupled receptors to stimulate the pro-inflammatory cascade after tissue injury, sensitize neurons to pain, stimulate smooth muscle contraction, regulate vasodilation, regulate temperature, and control ionic balance in the kidney [55]. In invertebrates, prostaglandins play similar roles as modulators of ion transport and regulators of immune response and reproduction [25,56]. In marine systems, they are most notable for their role as feeding deterrents; for example, ingestion of food laced with prostaglandins causes regurgitation and learned aversion in reef predators [57,58].

**Figure 8:**
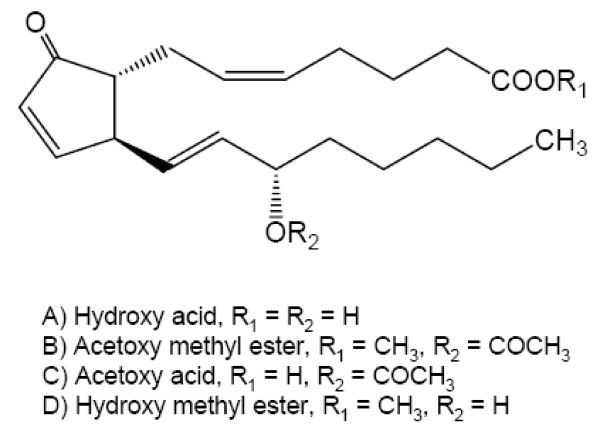
**Structure of prostaglandin A_2 _(PGA_2_) analogs from the gorgonian coral, *Plexaura homomalla***.

Pawlik and Fenical (1989) traced the anti-predatory properties of *P. homomalla *to the acetoxy acid, hydroxy methyl ester and hydroxy acid (0.2% of dry weight) of PGA_2 _(Figure 8), yet found no evidence for a deterrent effect of the abundant, fully-esterified form of PGA_2 _(averaging 1-2% dry weight) [52-54]. Chemical characterization of extracts from all seven gorgonian species used in the present study indicated that the hydroxy acid of PGA_2 _was present only in *P. homomalla *tissue, where it occurred at 0.06% dry weight [31]. Although the fully-esterified form of PGA_2 _may not act directly as a feeding deterrent [59], esterified prostaglandins may function as sources of activated chemical defenses, whereby damage to the coral may cause the innocuous acetoxy methyl esters to undergo lipase-mediated enzymatic hydrolysis to the noxious hydroxy acids over a period of several hours [52,59,60]. For less mobile predators, like *C. gibbosum*, the feeding-stimulated formation of the deterrent hydroxy acids of PGA_2 _in *P. homomalla *and/or acidic hydrolysis of esterified PGA_2 _forms in the low-pH gastric environment could pose a significant threat unless these obligate predators possessed orchestrated detoxification mechanisms capable of responding to host allelochemicals.

Within the diverse P450 superfamily, family 4 monooxygenases (CYP4) contain the major fatty acid ω-hydroxylases capable of preventing lipotoxicity [24]. However, the induction of CYP transcripts as a result of consuming *P. homomalla *did not extend to all *Cyphoma *CYP4 forms. The transcripts induced in response to *P. homomalla *dietary exposure (CYP4BL and CYP4BK) were most closely related to vertebrate CYP4A and CYP4F forms that are well known for their ability to metabolize prostaglandins, including the predominant prostaglandin (PGA_2_) in *P. homomalla *[61-64]. In most cases, induced transcripts within the CYP4BK and CYP4BL subfamily encode proteins that share amino acid residues determined to be important in prostaglandin metabolism by vertebrate prostaglandin hydroxylases (see Additional file 10). Moreover, CYP4V10 transcripts were not induced, nor would this have been expected, since this sequence falls within the CYP4C/V clade containing CYP forms whose roles have been postulated to include hormone-stimulated fatty acid oxidation during starvation (cockroach, CYP4C1) [65] and fatty acid metabolism in the retina in vertebrates (reviewed in [24]). The induction of CYP4BK and CYP4BL transcripts coincided with increased metabolism of the diagnostic eicosanoid LTB_4 _in the digestive gland of snails feeding on *P. homomalla*. Yeast-mediated expression of selected full-length *Cyphoma *CYP4BK and CYP4BL cDNAs demonstrated that both CYP4BL1 and CYP4BL3 proteins were capable of LTB_4 _hydroxylase activity. Moreover, CYP4BL3 is included in the subset of CYP4BL transcripts, represented by CYP4BL_(subA)_, that exhibited significant induction (5.1-fold) in snails feeding on *P. homomalla*. Together, the enzymatic activity and transcript data suggest that the CYP4BL3 protein may be responsible for the bulk of the observed eicosanoid hydroxylase activity in *Cyphoma *digestive gland. Collectively, these results provide the first indication that proteins in the CYP4 family in *Cyphoma *likely function in the metabolism of eicosanoids - possibly prostaglandins - and that these proteins may play an important role in the maintenance of specific marine consumer-host relationships.

The structurally diverse pool of prostaglandin forms (PGA_2_, PGE_2_, PGF_2α_) and their analogues found in *P. homomalla *tissues [52,66-70] may have necessitated the evolution of a variety of prostaglandin-metabolizing CYPs, resulting in the diversity of closely related CYP4BL transcripts identified here. Such diversity of CYP genes is thought to be a mechanism of adaptation in insects that consume allelochemically variable diets [12]. For example, one recent study suggested that the loss of host plant specialization in the insect *Papilio *(insect) lineage, favoring generalists like *P. glaucus*, may have evolved from only a small number of mutational changes within the SRS-6 region of CYP6B enzymes, allowing for the acquisition of novel catalytic activities while still retaining the ancestral furanocoumarin-metabolizing capability [71]. This gradual accumulation of functionally significant replacements, often following a series of gene duplications [72], has been hypothesized as a means for enzymes to evolve in response to host selection [71]. Many of the amino acid differences (22/40) among members of the CYP4BL group occur within or near the putative six SRS regions responsible for defining the range of possible substrates for cytochromes P450. Based on the location of these differences and knowledge gained from site-directed mutagenesis studies, it is likely that these variations will confer differences in catalytic activity and substrate specificity among these closely related proteins. Moreover, it would be interesting to explore whether the co-occurring specialist *Cyphoma signatum*, which feeds exclusively on *Plexaurella *spp.--known only to contain the eicosanoid 11*R*-hydroxy-5*Z*,8*Z*,12*E*,14*Z*-eicosateraenoic acid (11*R*-HETE) [73] and not prostaglandins--possesses reduced diversity of CYP4 forms capable of responding to foreign dietary prostaglandins.

The expansion of the CYP4BL cluster relative to the other *Cyphoma *CYPs suggests that positive selection may be acting to enhance the diversity of this subfamily, likely through repeated gene duplication and divergence, possibly tempered by concerted evolution involving gene conversion among clustered CYP4 genes. In addition, alternative splicing of transcripts or the formation of chimeric CYPs from the splicing of pre-mRNA molecules can allow for the generation of novel enzymes with divergent catalytic functions. There is evidence for both of these scenarios within the P450 superfamily. For example, the human CYP4F3 gene has two inducer-specific 5' UTR transcriptional start sites capable of controlling the mutually exclusive splicing of either exon 3 or 4, generating the tissue-specific expression of catalytically distinct CYP4F3A and CYP4F3B isoforms [74]. In addition, chimeric RNA molecules in which exons are joined together from distinct pre-mRNAs have been identified for both CYP2C and CYP3A members [75,76]. Formation of hybrid mRNA, especially by trans-splicing, allows detritus or solo exons to become functional even if they are dispersed in the genome [77]. In both cases, exon shuffling or alternative splicing of distinct transcripts provides the opportunity for novel catalytic functions to emerge, further increasing the diversity of cytochrome P450 genes. It is conceivable that one or both of these processes has occurred in *Cyphoma*. However, without specific knowledge of the genomic arrangement or exon-intron structure of *Cyphoma *CYP4 genes, we cannot distinguish between these and other possibilities. Nevertheless, the results of our studies suggest that further research on the structure of *Cyphoma *CYP4 genes and the catalytic function of their encoded proteins could provide insight into the evolution of CYP gene diversity and its role in allelochemical tolerance.

The CYP4 proteins identified in *Cyphoma *digestive gland are likely apart of a larger suite of inducible and constitutively active detoxification enzymes involved in the protection of this consumer from dietary intoxication. Cyclopentenone prostaglandins including PGA_2 _have been shown to be inducers and substrates of additional detoxification mechanisms, such as glutathione *S*-transferases (GST), whose activity has previously been linked to allelochemical tolerance in marine consumers [31,78,79]. GSTs are highly expressed in the digestive gland of *Cyphoma *[29], and a screening of gorgonian lipophilic extracts suggests that this consumer's gorgonian diet contains GST substrates [31]. Thus, GSTs may contribute to the detoxification of prostaglandins and other allelochemicals in *Cyphoma*. In addition, glutathione conjugates of prostaglandins and other eicosanoids are known to be effluxed by multidrug resistance-associated protein (MRP), and *Cyphoma *may likewise enlist the aid of these transporters to cope with its toxic diet [80]. It is quite possible that marine consumers that regularly exploit a range of allelochemically-rich prey may have evolved an equally diverse array of detoxification mechanisms.

## Conclusion

In summary, we provide several lines of evidence that implicate inducible *Cyphoma *CYP4s in the mechanism of adaptation to gorgonian allelochemicals. The corresponding pattern of transcriptional responsiveness and eicosanoid hydroxylase activity in the digestive gland of snails feeding on *P. homomalla *coupled with similarity between transcriptionally-active CYP4s and well-characterized fatty acid hydroxylases strongly indicates a role for the *Cyphoma *CYP4s in mediating the metabolism of dietary eicosanoids. This work demonstrates the utility of incorporating a pharmacological approach in ecological studies in order to better understand the biochemical innovations that allow marine consumers to tolerate allelochemically-defended prey. Moreover, identifying the molecular underpinnings of organismal physiological response has broad implications for understanding the role of the environment in determining gene function in a co-evolutionary context.

## Authors' contributions

KEW conducted the feeding assays, nucleic acid isolations, cloning, qRT-PCR analysis, recombinant expression of molluscan proteins in yeast, and drafted the manuscript. VRS assisted in the statistical analysis. DRN assisted in the phylogenetic analysis and naming of the novel CYPs. JVG performed the phylogenetic analysis and homology modeling. MEH assisted in the design of experiments, in data interpretation and in revising the manuscript. All authors read and approved the final manuscript.

## Supplementary Material

Additional file 1 **Summary of digestive gland RNA and protein samples collected during January 2006 feeding assays**. Click here for file

Additional file 2 **RNA extraction and cDNA synthesis**. Click here for file

Additional file 3 **Degenerate and specific oligonucleotide primers for initial and full-length amplification of *Cyphoma gibbosum *CYP4s and actin**. Click here for file

Additional file 4 **Real-time quantitative PCR methods and statistical analysis**. Click here for file

Additional file 5 **Oligonucleotide primers for quantitative PCR experiments**. Click here for file

Additional file 6 **Maximum parsimony trees depicting transcripts recognized by CYP4BL_(subA) _and CYP4BL_(subB) _quantitative RT-PCR primers**. Click here for file

Additional file 7 **Recombinant expression of *Cyphoma *CYPs in *Saccharomyces *cerevisiae**. Click here for file

Additional file 8 **Oligonucleotide primers for recombinant *C. gibbosum *CYP4 expression in yeast**. Click here for file

Additional file 9 **Enzymatic analysis**. Click here for file

Additional file 10 **Comparison of CYP4 protein sequences**. Click here for file

Additional file 11 **Phylogenetic analysis of metazoan CYP4s**. Click here for file

Additional file 12 **Results of a one-way MANOVA investigating CYP4 gene expression variability among reefs for time-zero and control-fed *C. gibbosum***. Click here for file

Additional file 13 **Results of a two-way MANOVA investigating differences in digestive gland CYP4 gene expression in *C. gibbosum *feeding on control versus gorgonian diets**. Click here for file

Additional file 14 **Results of ANOVA comparisons (Univariate F-tests) of diet- and reef-specific mean CYP4 gene expression in *C. gibbosum *feeding on control vs. gorgonian diets**. Click here for file

Additional file 15 **Leukotriene B_4 _hydroxylase activity in *Cyphoma *microsomes**. Click here for file

Additional file 16 **Leukotriene B_4 _hydroxylase activity of heterologously expressed *Cyphoma* CYP4 proteins**. Click here for file

Additional file 17 **Homology models CYP4BL3 and CYP4BK1 depicting putative substrate access channels**. Click here for file

Additional file 18 **Deduced amino acid alignment of *Cyphoma *CYP4V, CYP4BK and CYP4BL consensus sequences**. Click here for file
