# Supplementary material for: Cytochrome P450 diversity and induction by gorgonian allelochemicals in the marine gastropod Cyphoma gibbosum
Source: BMC Ecol. 2010 Dec 1;10:24. doi: 10.1186/1472-6785-10-24 (PMC3022543; doi:10.1186/1472-6785-10-24)
Supplement: Additional file 1 — Summary of digestive gland RNA and protein samples collected during January 2006 feeding assays. [file 1472-6785-10-24-S1.PDF]

Additional file 1. Summary of digestive gland RNA and protein samples collected during January 2006 feeding assays

	Reef											
	Big Point		North Normans		Rainbow Gardens		Shark Rock		Sugar Blue Holes		**Total**	
Diet group	RNA	Protein	RNA	Protein	RNA	Protein	RNA	Protein	RNA	Protein	**RNA**	**Protein**
B. asbestinum	2	1	2	0	5	1	2	1	2	1	**13**	**4**
E. mammosa	2	1	2	0	4	1	2	1	2	1	**12**	**4**
G. ventalina	2	1	2	0	5	1	2	1	2	1	**13**	**4**
P. acerosa	2	1	2	0	2	1	2	1	2	1	**10**	**4**
P. americana	2	1	2	0	3	2	3	2	2	2	**12**	**7**
P. elisabethae	2	0	2	0	0	0	0	0	2	0	**6**	**0**
P. homomalla	2	1	2	0	3	1	2	1	2	1	**11**	**4**
Control	6	3	6	0	9	3	6	3	6	3	**33**	**12**
Time Zero	6		6		6		6		7		**31**	

Numbers reflect the total number of digestive glands collected in each category.
