# Supplementary material for: Cytochrome P450 diversity and induction by gorgonian allelochemicals in the marine gastropod Cyphoma gibbosum
Source: BMC Ecol. 2010 Dec 1;10:24. doi: 10.1186/1472-6785-10-24 (PMC3022543; doi:10.1186/1472-6785-10-24)
Supplement: Additional file 2 — RNA extraction and cDNA synthesis. [file 1472-6785-10-24-S2.PDF]

Additional file 2. RNA extraction and cDNA synthesis

Initial cDNA cloning

Total RNA was isolated from pooled digestive glands from fifteen snails collected from the following reefs Big Point, North Normans, Shark Rock, and Windsock reef using RNA STAT-60 (Tel-Test B, Inc., Friendswood, TX) according to the manufacturer's protocol. Poly(A)+ RNA was then purified using the MicroPoly(A)Purist mRNA purification kit (Ambion, Austin, TX) according to the manufacturer's instructions. First-strand cDNA was reverse transcribed from 2 μg poly(A)+ RNA using OmniScript reverse transcriptase (OmniScript RT kit, Qiagen) with random hexamer primers.

Degenerate primers were designed to conserved amino acid sequences within the CYP4 P450 fingerprint region. PCR was performed using AmpliTaq Gold DNA polymerase (Applied Biosystems) under the following conditions: 94°C for 10 min; 35 cycles of 94°C for 15 sec, 52°C for 30 sec; 72°C for 7 min with pairs of degenerate primers (CYP4_F1/ CYP4_R3; CYP4_F2/ CYP4_R3; see Additional file 3 for all primers used). PCR products were visualized on agarose gels, gel purified using a Geneclean® kit (Qbiogene, Irvine, CA), ligated into pGEM-T Easy© plasmid vector (Promega, Madison, WI), and transformed into JM109 cells (Promega). Cloned products were sequenced in both directions using an ABI 3730XL capillary sequencer at the Keck facility of the Josephine Bay Paul Center for Comparative Molecular Biology & Evolution at the Marine Biological Laboratory (Woods Hole, MA). A minimum of twelve clones were sequenced for each PCR fragment. Clones were initially clustered based on nucleotide sequence identity (>80%) with Sequencher 4.6 (Gene Codes Corp., Ann Arbor, MI) and a consensus sequence was generated and examined by NCBI/GenBank BLASTx for gene identification (Altschul *et al.*, 1997). All PCR primers were obtained from Sigma Genosys (St. Louis, MO).

ALTSCHUL, S. F., MADDEN, T. L., SCHAFFER, A. A., ZHANG, J., ZHANG, Z., MILLER, W. & LIPMAN, D. J. (1997) Gapped BLAST and PSI-BLAST: a new generation of protein database search programs. *Nucleic Acids Research,* **25**, 3389-3402.
