# Supplementary material for: Cytochrome P450 diversity and induction by gorgonian allelochemicals in the marine gastropod Cyphoma gibbosum
Source: BMC Ecol. 2010 Dec 1;10:24. doi: 10.1186/1472-6785-10-24 (PMC3022543; doi:10.1186/1472-6785-10-24)
Supplement: Additional file 3 — Degenerate and specific oligonucleotide primers for initial and full-length amplification of Cyphoma gibbosum CYP4s and actin. [file 1472-6785-10-24-S3.PDF]

Additional file 3. Degenerate and specific oligonucleotide primers for initial and full-length amplification of *Cyphoma gibbosum* CYP4s and actin

Gene(s)	Primers	Orientation	Sequences (5' to 3')	Application
CYP4	CYP4_F1	Forward	GAY ACI TTY ATG TTY GAR GG	Initial amplification of partial cDNAs
	CYP4_F2	Forward	ATG TTY GAR GGI CAY GAY ACI AC	
	CYP4_R3	Reverse	GCR ATY TTY TGI CCI ATR CAT TT	
Adaptor primers	RACE_1_F		AAT ACG ACT CAC TAT AGG	Adaptor Primers for RACE
	AP1		CCA TCC TAA TAC GAC TCA CTA TAG GGC	
CYP4-1[a]	CYP4_F11_R	Reverse	TGA CGT GAA GAC TAT CAC TGT GAC	5' RACE
CYP4-2[a]	CYP4_A12_R	Reverse	GAA GAT ATG GAT CGT ACA TAC CG	5' RACE
	CYP4-2_F1	Forward	GAG AAG TTT TCA GCA ACA TGG ACT TGG	Full-length cloning of CYP4-2 group
	CYP4-2_R1	Reverse	GGA AGA TCA CCG TGT GTG AAA GGA C	
CYP4-3[a]	CYP4_D09_F	Forward	ACC TTG TAC AGC CTG GCG AGA TGG	3' RACE
	CYP4_D09_R	Reverse	CCA AAT ATC TAT TGC TAT GAA TG	5' RACE
	CYP4-3_R1	Reverse	AGT GGC CAA TGG AGC ACG TAG G	5' RACE
	CYP4-3_R2	Reverse	GCA GGT AGT CAT GTG GCC TGT C	5' RACE
	CYP4-3_F3	Forward	ACA AGA CGT TTC GAC AGA ACA GTT AGC	Full-length cloning of CYP4-3 group
	CYP4-3_R6	Reverse	TTG CTG TCC CAC AGT GTC AAC AAG	
Actin	DegAct5	Forward	ACA ACG GYT CSG GYA TGT GC	Degenerate actin primers
	DegAct3	Reverse	GAA GCA YTT GCG RTG WAC RAT	

[a] Transcripts within the following groups (CYP4-1, CYP4-2 and CYP4-3) have been subsequently assigned to the CYP4V10, CYP4BK, and CYP4BL subfamilies, respectively.
