# Supplementary material for: Cytochrome P450 diversity and induction by gorgonian allelochemicals in the marine gastropod Cyphoma gibbosum
Source: BMC Ecol. 2010 Dec 1;10:24. doi: 10.1186/1472-6785-10-24 (PMC3022543; doi:10.1186/1472-6785-10-24)
Supplement: Additional file 4 — Real-time quantitative PCR methods and statistical analysis. [file 1472-6785-10-24-S4.PDF]

Additional file 4. Real-time quantitative PCR methods and analysis

Real-time quantitative PCR conditions

The PCR conditions were as follows: 95°C for 3min; 40 cycles of 95°C for 15 sec, 64°C or 62°C for 1 min. PCR product specificity from each primer pair was confirmed by melt curve analysis to ensure that only a single product was amplified. Additionally, a representative group of ten poly(A)+ RNA samples was used to perform control cDNA synthesis experiments without reverse transcriptase to check for contamination by genomic DNA. For each RNA sample, relative transcript abundance was calculated from a standard curve that was generated for each qPCR primer set by serially diluting plasmids containing the fragment to be amplified. Each sample and standard was run in duplicate and the expression of *C. gibbosum* β-actin was used to control for differences in cDNA synthesis among samples.

Statistical analysis

To examine whether cytochrome P450 transcript expression differed between snails feeding on a control diet versus each gorgonian diet, a two way multivariate analysis of variance (MANOVA) was used with Diet (control vs. gorgonian diet) as a fixed factor and Reef (snail origin) as a random factor. If Diet was found to be significant in the two-way MANOVA, a univariate two-way analysis of variance (ANOVAs) was used to determine which CYP4 grouping(s) were significantly different. In addition, a one-way MANOVA was used to examine the variability in CYP4 transcript expression among reefs, where Reef was considered a random factor. This test was used to investigate reef-specific variation in transcript levels in the time-zero group snails, and to determine if any such variation persisted in snails collected from these same reefs after being fed a control diet for four days. For MANOVA and ANOVA analyses the dependent variable, the relative transcript number for each CYP4 grouping, was log transformed prior to statistical analysis to homogenize variances. Data analysis was performed using SYSTAT® version 11 (Systat Software, Inc., San Jose, CA) and P-values were corrected for both MANOVA analyses using Bonferroni's adjustment.
