# Supplementary material for: Cytochrome P450 diversity and induction by gorgonian allelochemicals in the marine gastropod Cyphoma gibbosum
Source: BMC Ecol. 2010 Dec 1;10:24. doi: 10.1186/1472-6785-10-24 (PMC3022543; doi:10.1186/1472-6785-10-24)
Supplement: Additional file 5 — Oligonucleotide primers for quantitative PCR experiments. [file 1472-6785-10-24-S5.PDF]

Additional file 5. Oligonucleotide primers for quantitative PCR experiments

Gene target[a]	Primers	Direction	Sequence (5' to 3')
CYP4V10	F11_58_23_ext	Forward	GCT GAC TCC AAC TTT CCA CT
	R3_58_23_ext	Reverse	GGC CCT TAT CTA CAT GAT GC
CYP4BK	F6_60_1_ext	Forward	GGG AGA TCT GCA CAA GCT CC
	R15_60_1_ext	Reverse	GAA AGG GCC GAG CCA AAA GC
CYP4BL	197_all_F1	Forward	TTT GCC ATG AAT GAA TTG AAG GTG
	197_all_R1	Reverse	GGA GCA CGT AGG ATT TCA TGA TTT GG
$CYP4BL_{(sub\ A)}$	F12_54_9_ext	Forward	ATG CCC TGT TAC AGG GAC GT
	R11_54_9_ext	Reverse	GTC CGT CCA GGT TCA AAG GT
$CYP4BL_{(sub\ B)}$	F1_54_4_ext	Forward	TGA TCT GAC TCA GCT GCC ATA C
	R1_54_4_ext	Reverse	CTC TGT GAT TTC TCG CTC AAT G
Actin	F3_CgActin	Forward	TCG GTC GTC CCA GAC ATC AGG
	R1_actinRT	Reverse	TCT CCA TGT CGT CCC AGT TGG TG

[a] $CYP4BL_{(sub\ A)}$ and $CYP4BL_{(sub\ B)}$ detect select sequences within the CYP4BL subfamily. $CYP4BL_{(sub\ A)}$ primers detect clones representing the following genes: CYP4BL2 thru 4. $CYP4BL_{(sub\ B)}$ primers detect clones representing the following genes: CYP4BL1, CYP4BL2, and CYP4BL5 thru 9.
