# Supplementary material for: Cytochrome P450 diversity and induction by gorgonian allelochemicals in the marine gastropod Cyphoma gibbosum
Source: BMC Ecol. 2010 Dec 1;10:24. doi: 10.1186/1472-6785-10-24 (PMC3022543; doi:10.1186/1472-6785-10-24)

Additional file 6. Maximum parsimony trees depicting transcripts recognized by CYP4BL$_{(subA)}$ and CYP4BL$_{(subB)}$ quantitative RT-PCR primers. In part (A) the unrooted maximum parsimony tree shows 168 clones partitioned into six clusters representing nine putative CYP4BL genes. In each cluster the number of clones recognized by primer set CYP4BL$_{(sub\ A)}$, CYP4BL$_{(sub\ B)}$ and those not recognized (nd) by either set are indicated. The unrooted maximum parsimony tree was further transformed (cladogram) into a circular tree using FigTree v1.1.2 (Rambaut 2007) in part (B). The color of the node indicates the qPCR primer set used for detection. Clones containing two or less mismatches not within ten base pairs of the 3' end were considered to be recognized by their respective primer set.

Rambaut A (2007) Fig Treev1.1.2. Edinburgh (UK): Institute of Evolutionary Biology, University of Edinburgh.

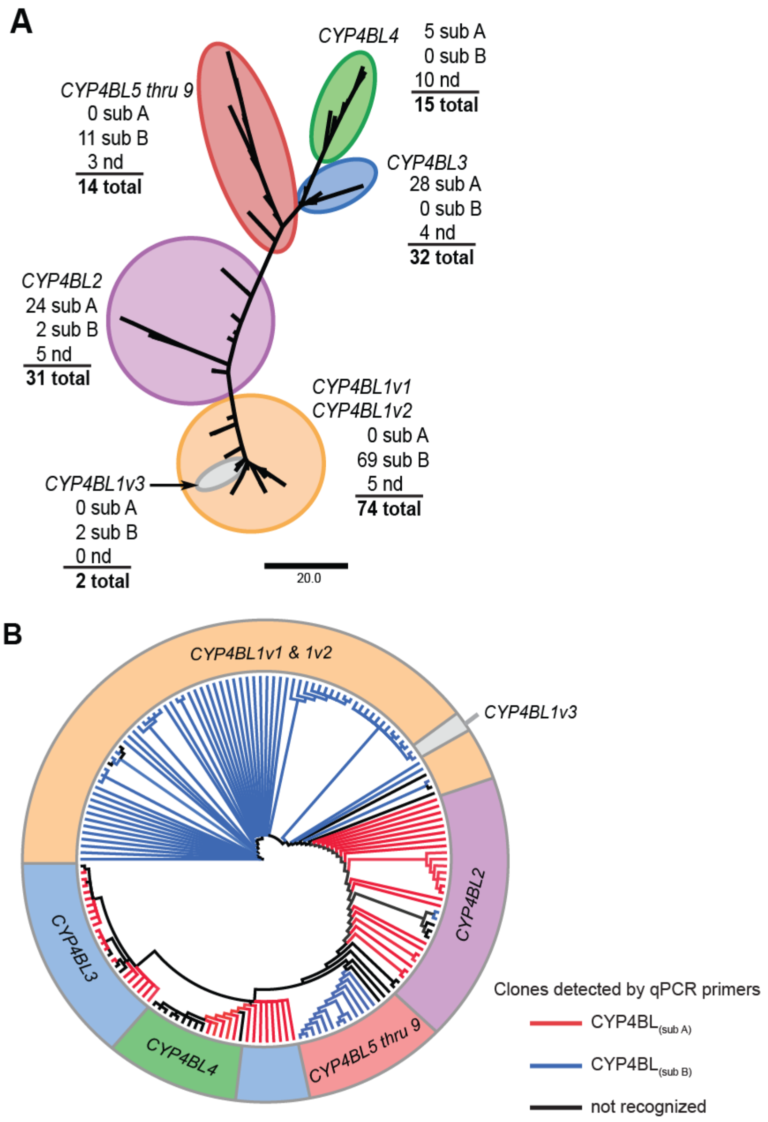

Supplement: Additional file 6 — Maximum parsimony trees depicting transcripts recognized by CYP4BL(subA) and CYP4BL(subB) quantitative RT-PCR primers. [file 1472-6785-10-24-S6.PDF]
