# Supplementary material for: Cytochrome P450 diversity and induction by gorgonian allelochemicals in the marine gastropod Cyphoma gibbosum
Source: BMC Ecol. 2010 Dec 1;10:24. doi: 10.1186/1472-6785-10-24 (PMC3022543; doi:10.1186/1472-6785-10-24)
Supplement: Additional file 7 — Recombinant expression of Cyphoma CYPs in Saccharomyces cerevisiae. [file 1472-6785-10-24-S7.PDF]

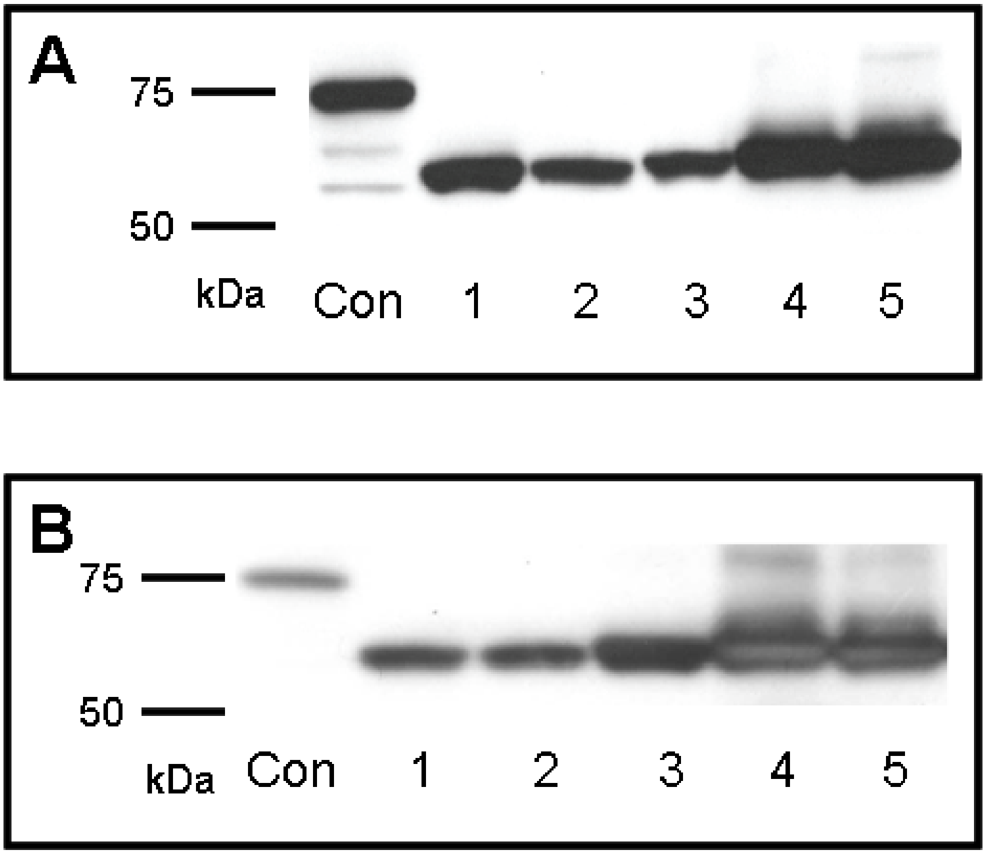

Additional file 7. Recombinant expression of *Cyphoma* CYPs in *Saccharomyces* cerevisiae. The W(R) strain of yeast was transformed with *Cyphoma* CYPs under the control of a galactose-inducible promoter. After 8 hours (A) or 15 hours (B) of induction in YPGE media containing 2% galactose, CYP expression was visualized by Western blotting with anti-V5-HRP (Invitrogen). Each lane contains 20 µg of protein. Lanes: Con, positive control gene expression *Arabidopsis* β-glucuronidase (75 kDa); lane 1, CYP4BK1; lane 2, CYP4BK2; lane 3, CYP4BL1; lane 4, CYP4BL4; lane 5, CYP4BL3. *Cyphoma* CYP4 proteins are estimated to be 64 kDa based on experimental calculations of their deduced amino acid sequence.
