# Supplementary material for: Cytochrome P450 diversity and induction by gorgonian allelochemicals in the marine gastropod Cyphoma gibbosum
Source: BMC Ecol. 2010 Dec 1;10:24. doi: 10.1186/1472-6785-10-24 (PMC3022543; doi:10.1186/1472-6785-10-24)
Supplement: Additional file 8 — Oligonucleotide primers for recombinant C. gibbosum CYP4 expression in yeast. [file 1472-6785-10-24-S8.PDF]

Additional file 8. Oligonucleotide primers for recombinant *C. gibbosum* CYP4 expression in yeast

Gene	Clone	Primers	Direction	Sequence (5' to 3')
CYP4BK1	198_58	pENTR_198_27_F1	Forward	CAC CAT GGA CTT GGG TTT TTC CTC C
		pENTR_198_58_R1	Reverse	TAC AGT GGG TGT TCT TGG AGT GGC AAA CAT
CYP4BK2	198_27	pENTR_198_27_F1	Forward	CAC CAT GGA CTT GGG TTT TTC CTC C
		pENTR_198_27_R1	Reverse	ATG TTC CCA GTT GTT ATA TAC AGT GGG TGT
CYP4BL1	197_52	pENTR_197_53_F1	Forward	CAC CAT GGA TGA TAC CTT CTC TCA ACT G
CYP4BL3	197_53	pENTR_197_53_R1	Reverse	GCC TTT GCG TGG CGT GAT
CYP4BL4	197_48			
