# Supplementary material for: Cytochrome P450 diversity and induction by gorgonian allelochemicals in the marine gastropod Cyphoma gibbosum
Source: BMC Ecol. 2010 Dec 1;10:24. doi: 10.1186/1472-6785-10-24 (PMC3022543; doi:10.1186/1472-6785-10-24)
Supplement: Additional file 9 — Enzymatic analysis. [file 1472-6785-10-24-S9.PDF]

Additional file 9. Enzymatic analysis

P450 content and reductase activity

Cytochrome P450 specific content of yeast and selected digestive gland microsomal samples was determined spectrophotometrically using the carbon monoxide difference spectrum ($\Delta OD_{450\text{-}490}$) of sodium dithionite-reduced samples (1 mg/mL) and an extinction coefficient of 91 $mM^{-1}cm^{-1}$ by the method of (Omura and Sato, 1964). P450 reductase activity in both selected yeast and digestive gland microsomal samples was determined by measuring NADPH-cytochrome *c* reductase activity using the rate of cytochrome *c* reduction calculated from the increase in absorbance at 550 nm ($\Delta\varepsilon_{550}$ 21.1 mM^{-1} cm^{-1}) using a UV-2401PC spectrophotomer (Shimadzu). The reaction mixture (in a final volume of 200 µL) contained 0.2 M potassium phosphate buffer, pH 7.7, 80 µM equine heart type III Cytochrome *c* (Sigma), and 0.2 mM NADPH. One unit of reductase activity is defined as the amount of enzyme which can reduce 1 nmol of cytochrome *c* per min.

The characteristic P450 spectral peak was not detected in *Cyphoma* digestive gland microsomal preparations nor was there a peak at 420 nm, which would have indicated the presence of degraded protein (data not shown). Cytochrome P450 was consistently detected in recombinant microsomes from yeast induced for 8 hrs on galactose-containing media (27.4 pmol P450 mg $protein^{-1}$ – 118.7 pmol P450 mg $protein^{-1}$), but not in yeast induced for 15 hrs. No 420 nm peak was ever observed in any yeast microsomal preparations. The lack of chromophore detection at 450 nm with spectrophotometry in some yeast samples does not preclude NADPH-dependent enzymatic activities in yeast microsomes (Bylund and Oliw, 2001). NADPH-cytochrome P450 reductase activity was also confirmed to be highly expressed in all galactose-induced yeast microsomal samples (~ 5000 nmol min^{-1} mg $protein^{-1}$) and in the one snail digestive gland examined (631 nmol min^{-1} mg $protein^{-1}$).

Lauric acid hydroxylase activity

Yeast microsomes were incubated for 80 min at 25°C and human liver microsomes (1.0 mg/mL) were incubated for 10 min at 37°C. Negative control incubations consisting of the same reaction mixture without microsomal proteins were also performed. All samples were run in single determinations. The reactions were stopped by the addition of 50 µL of 94% acetonitrile/6% acetic acid, centrifuged at 10,000 x g for 3 min, and 75 µL of supernatant was injected onto a reverse phase Agilent Zorbax C18 HPLC column (5 µm, 4.6 mm x 250 mm). The metabolites were separated at 45°C with methanol/acetonitrile/water (23:23:54) containing 1 mM perchloric acid adjusted to 100% methanol over 35 min with a flow rate of 1.0 mL/min. Lauric acid and the ω-hydroxylated metabolite were detected by liquid scintillation counting. Control reactions contained the same components as the samples, but were terminated at the start of the incubation. Lauric acid was not metabolized to any detectable products by the yeast microsomes.

Leukotriene B_4 hydroxylase activity

Cyphoma microsomes were incubated for 60 min at 30°C; yeast and human (0.5 mg/mL) microsomes were incubated for 120 min at both 23°C and 30°C. Reactions were terminated after 120 mins by the addition of 25 µL of 94% acetonitrile/6% glacial acetic acid. Incubations were centrifuged at 10,000 x g for 3 min. and 80 µL of the supernatant was injected onto a reverse phase Agilent Zorbax C18 HPLC column (5µm, 4.6 mm x 250 mm) and separated with an initial mobile phase consisting of 30% acetonitrile with 1 mM perchloric acid in water changing to 70% methanol over 20 minutes at a flow rate of 1.0 mL/min. Products were detected by monitoring absorbance at 270 nm and compared to known standards. All samples were run in duplicate. Control reactions without microsomal protein, without NADPH and without incubation were performed with two "global control" samples made by either pooling all of the yeast microsomal samples or the snail microsomal samples.

Only those *Cyphoma* microsomes isolated from the digestive glands of snails feeding on the control diets (n = 10) and *P. homomalla* (n = 4) were analyzed for LTB_4 activity. This decision was made based the fact that CYP4 gene induction was only observed in individual snails feeding on *P. homomalla*, and cost considerations associated with sample analysis.

All lauric acid and leukotriene B_4 hydroxylase assays were performed by BD Biosciences Discovery Labware Division, Woburn, MA under the supervision of Dr. David Stressor.

BYLUND, J. & OLIW, E. H. (2001) Cloning and characterization of CYP4F21: a prostaglandin E2 20-hydroxylase of ram seminal vesicles. *Archives of Biochemistry and Biophysics,* 389**,** 123-129.

OMURA, T. & SATO, R. (1964) The carbon monoxide-binding pigment of liver microsomes. *Journal of Biological Chemistry,* 239**,** 2370.
