# Supplementary material for: Cytochrome P450 diversity and induction by gorgonian allelochemicals in the marine gastropod Cyphoma gibbosum
Source: BMC Ecol. 2010 Dec 1;10:24. doi: 10.1186/1472-6785-10-24 (PMC3022543; doi:10.1186/1472-6785-10-24)
Supplement: Additional file 10 — Comparison of CYP4 protein sequences. [file 1472-6785-10-24-S10.PDF]

Additional file 10. Comparison of CYP4 protein sequences

Sequence analysis

Maximum parsimony analysis identified one CYP4V10 gene with two putative allelic variants (CYP4V10v1 and CYP4V10v2) that share 98.8% amino acid identity (Fig. 4, part A) but differ by a five amino acid insertion near their C-terminus end. Five of the 65 clones within the CYP4V10 subfamily also contained a 68 bp deletion causing a frame shift, resulting in a premature stop codon and a loss of 155 amino acids, likely resulting in a nonfunctional P450 protein lacking its heme-binding region.

Within the CYP4BK subfamily, there were two distinct genes (CYP4BK1 and CYP4BK2) sharing 93.5% amino acid identity (Fig. 4, part B); in addition to the differences in specific residues, CYP4BK1 and CYP4BK2 differ from one another by the addition of six amino acids at the C-terminus of CYP4BK2. Both the CYP4BK1 and CYP4BK2 cDNA sequences contain the nucleotides encoding this additional sequence; however, a mutation in CYP4BK1 has lead to a stop codon truncating the protein. The highly similar 3' UTRs suggest that CYP4BK1 and CYP4BK2 arose from a recent duplication event, followed by a diversification of both forms; however, which sequence is representative of the ancestral form is unknown. The unrooted maximum parsimony tree also suggests that CYP4BK2 likely has two allelic variants (Fig. 4, part B).

The CYP4BL subfamily was the most diverse. From analysis of tree topologies and pairwise comparisons of cDNA sequences we inferred the possible existence of nine distinct loci (Fig. 4, part C) encoding proteins sharing between 89.2 and 99.2% amino acid identity. The CYP4BL1 gene is represented by three apparent allelic variants (CYP4BL1v1, CYP4BL1v2, CYP4BL1v3), which share >99% amino acid identity when positions common to all three variants are compared. CYP4BL1v2 contains an in-frame deletion of 21 amino acids, resulting in a protein lacking the majority of the F-helix and a portion of the substrate recognition site (SRS) 2. With the exception of this deletion, the protein coding sequences of CYP4BL1v1 and CYP4BL1v2 are identical. CYP4BL1v3 is characterized by an addition of thirteen amino acids at the C-terminus resulting from a single nucleotide substitution in the corresponding stop codon in allelic variants 1 and 2.

The remaining members of the CYP4BL family (CYP4BL2-CYP4BL9), are predicted to share between 94.2 to 99.2% amino acid identities (Fig. 4, parts C, D). Typically sequences having $\leq$ 3% divergence are designated as allelic variants (Nelson 2006). However, there are examples of distinct P450 genes sharing greater than 97% amino acid identity, further complicating allelic variant vs. putative paralogue assignment (Nebert et al. 1989). Furthermore, previous studies in the chemical ecology literature examining allelochemical-metabolizing CYPs have identified a multitude of closely related genes (92.8 to 99.8%) (Li et al., 2001), similar to the degree of sequence identity found in members of the CYP4BL subfamily in *Cyphoma* (94.2 to 99.2%). The authors suggested that the isolation of multiple closely related genes likely arose through repeated duplication and divergence from a single progenitor gene. While it remains possible that the CYP4BL subfamily consists of multiple allelic variants of a few genes rather than nine distinct loci, the large number of sequences obtained leads us to favor the latter. The extensive sequence variation and lack of clustering among clones within the CYP4BL5-9 group (Figure 4,

part D) made it difficult to generate a single consensus nucleotide sequence. In lieu of creating a consensus nucleotide sequence with no physical representatives, five full-length clones were designated as CYP4BL5-CYP4BL9 to illustrate the range of gene diversity. Pairwise sequence analyses among all CYP4BL sequences provided evidence for gene conversion and/or hybrid sequences (not shown). Thus, CYP4BL3, CYP4BL8, and CYP4BL9 are highly similar in the N-terminal halves, suggesting a cluster of genes that has undergone gene conversion. Similarly, CYP4BL9 shares extensive sequence identify with CYP4BL1 in the C-terminal halve. CYP4BL2 has an N-terminus nearly identical to that of CYP4BL1 and a C-terminus similar to that of CYP4BL3. Genomic characterization of *Cyphoma* CYP4 genes and gene clusters will be necessary for a definitive assessment of the number of loci and their relationships.

Identification of substrate access channels and active site residues

To obtain insight into the structural features of *Cyphoma* CYP4 proteins and make inferences about possible substrates, we created homology models of CYP4BL3 and CYP4BK1 and used them to determine whether they differ in their substrate access channels and/or catalytic sites. The CYP4BL3 model differed from CYP4BK1 in the absence of a third hydrophobic channel above the heme plane (see Additional file 16) due to the presence of an extended G and F loop occupying this space. Additionally, a bulky aromatic amino acid Trp226, located within the substrate recognition site (SRS) 2 of CYP4BL3 and extending toward the catalytic pocket, is lacking in CYP4BK1 yielding a catalytic site whose volume (2849.1 $Å^3$) is 20% less than that of CYP4BK1 (3445.6 $Å^3$); this may to a more restricted catalytic pocket (see Additional file 16).

Even though sequence similarity among members of the same subfamily is high for *Cyphoma* CYP4 proteins, variation in even one residue can have a profound effect on enzymatic function (Lindberg and Negishi, 1989). An alignment of the deduced amino acid sequences for both the CYP4BK and CYP4BL subfamilies indicated that 55% of the sequence variation within each subfamily (15 of 27 for CYP4BK subfamily and 22 of 40 for CYP4BL subfamily) falls within or near (within two residues) of the SRSs critical for defining the range of substrates metabolized by CYPs and described in Gotoh (1992) (see Additional file 17).

The potential importance of these sequence variations within the SRS in defining the substrate range and hydroxylation positions are highlighted by the two following examples. First, within the SRS2 region of mammalian CYPs, site-directed mutational analysis highlighted residues Y204 and F252 in rabbit CYP4A4 as being important in prostaglandin E_1 (PGE_1) hydroxylation (Loughran et al., 2000). Both of these residues are conserved in *Cyphoma* CYP4BK proteins. When these residues are replaced by H206 and S255, as seen in rabbit CYP4A7, metabolism of PGE_1 is decreased. *Cyphoma* CYP4BL proteins differ in the residues (either Y or H) present in the first position, but posses a tryptophan (W) in the second position, representing a conserved substitution from the aromatic phenylalanine (F) present in CYP4A4. Second, despite the modest overall sequence identity between *Cyphoma* CYP4 and human CYP4A proteins, all of the residues near the active site heme are fully conserved between human CYP4A11 and *Cyphoma* CYP4BK and CYP4BL subfamilies. An exception is *Cyphoma* CYP4BK2, which contains an alanine instead of a glutamate at position 313. Within the active site of mammalian CYP4A, CYP4B, and CYP4F forms, this glutamate residue binds to the heme and positions the fatty acid in a position favoring ω-hydroxylation (Stark et al., 2005). Mutating this glutamate to

an alanine results in the reduction or complete elimination of terminal carbon hydroxylation (ω-hydroxylation) (Dierks et al., 1998), and reduced fatty acid hydroxylation (Zheng et al., 2003). Thus, the presence of an alanine at this position may have contributed to the lack of fatty acid hydroxylase activity seen in *Cyphoma* CYP4BK2.

DIERKS, E. A., DAVIS, S. C. & ORTIZ DE MONTELLANO, P. R. (1998) Glu-320 and Asp-323 are determinants of the CYP4A1 hydroxylation regiospecificity and resistance to inactivation by 1-aminobenzotriazole. *Biochemistry,* 37**,** 1839-1847.

GOTOH, O., (1992) Substrate recognition sites in cytochrome P450 family 2 (CYP2) proteins inferred from comparative analyses of amino acid and coding nucleotide sequences. *Journal of Biological Chemistry*, 267, 83-90.

LI, W., BERENBAUM, M., and SCHULER, M.A. (2001) Molecular analysis of multiple CYP6B genes from polyphagus *Papilio* species, *Insect Biochemistry and Molecular Biology*, 31. 999-1011.

LINDBERG, R. L. P. & NEGISHI, M. (1989) Alteration of mouse cytochrome P450coh substrate specificity by mutation of a single amino-acid residue. *Nature,* 339**,** 632-634.

LOUGHRAN, P. A., ROMAN, L. J., AITKEN, A. E., MILLER, T. & MASTERS, B. S. S. (2000) Identification of unique amino acids that modulate CYP4A7 activity. *Biochemistry,* 39**,** 15110-15120.

NEBERT, D.W., NELSON, D.R., ADESNIK, M., COON, M.J., ESTABROOK, R.W., et al. (1989) The P450 Superfamily: Updated Listing of All Genes and Recommended Nomenclature for the Chromosomal Loci, *DNA*, 8, 1-13.

NELSON, D.R. (2006) Cytochrome P450 nomenclature, *Cytochrome P450 Protocols*, Humana Press, pp.1-10.

STARK, K., WONGSUD, B., BURMAN, R. & OLIW, E. H. (2005) Oxygenation of polyunsaturated long chain fatty acids by recombinant CYP4F8 and CYP4F12 and catalytic importance of Tyr-125 and Gly-328 of CYP4F8. *Archives of Biochemistry and Biophysics,* 441**,** 174-181.

ZHENG, Y.-M., BAER, B. R., KNELLER, B., HENNE, K. R., KUNZE, K. L. & RETTIE, A. E. (2003) Covalent heme binding to CYP4B1 via Glu310 and a carbocation porphyrin intermediate. *Biochemistry,* 42**,** 4601-4606.
