# Supplementary material for: Cytochrome P450 diversity and induction by gorgonian allelochemicals in the marine gastropod Cyphoma gibbosum
Source: BMC Ecol. 2010 Dec 1;10:24. doi: 10.1186/1472-6785-10-24 (PMC3022543; doi:10.1186/1472-6785-10-24)
Supplement: Additional file 11 — Phylogenetic analysis of metazoan CYP4s. [file 1472-6785-10-24-S11.PDF]

Additional file 18. Phylogenetic analysis of metazoan CYP4s

Addendum to Figure 6. Sequences include: Abalone CYP4V13 formally CYP4C17 (*Haliotis rufescens*, AAC32833); Anemone (*Nematostella vectensis*, JGI # 194368, JGI # 86714); Cockroach CYP4C7 (*Diploptera punctata*, AAC69184); Crayfish CYP4V11 formally CYP4C15 (*Orconectes limosus*, AAF09264); Fly CYP4C3 (*Drosophila melanogaster*, FlyBase FBpp0085074); Frog CYP4F42 (*Xenopus laevis*, BAD02914.1); Green crab CYP4V16 formally CYP4C39 (*Carcinus maenas*, JC8026); Hard clam CYP30 (*Mercenaria mercenaria*, AAB66556); Human CYP4A11 (NP_000769.2), CYP4A22 (NP_001010969.2), CYP4B1 (NP_000770.2), CYP4F11 (NP_067010.2), CYP4F12 (NP_076433.2), CYP4F2 (XP_001172541.1), CYP4F22 (NP_775754.2), CYP4F3 (NP_000887.2), CYP4F8 (NP_009184.1), CYP4V2 (NP_997235.2), CYP4X1 (NP_828847.1), CYP4Z1 (NP_835235.1); Limpet (*Lottia gigantea*, JGI# 122078, JGI# 154296, JGI# 178560, JGI# 206515, JGI# 211775, JGI# 237658); Mouse CYP4A12a (NP_803125.2), CYP4A12b (NP_758510.2), CYP4A14 (NP_031848.1), CYP4B1 (NP_031849.1), CYP4F13 (NP_570952.1), CYP4F14 (NP_071879.1), CYP4F15 (NP_598888.1), CYP4F16 (NP_077762.1), CYP4F18 (NP_077764.1), CYP4F37 (NP_001093657.1), CYP4F39 (AAI45756), CYP4F40 (NP_001095058.1); Mussel CYP4Y1 (*Mytilus galloprovincialis*, AAC32835); Polychaete worm (*Capitella sp*. JGI# 130691, JGI# 144012, JGI# 162759, JGI# 181976); Polychaete worm CYP4AT1 (*Capitella capitata,* AAS87604); Sand worm CYP4BB1 (*N. virens*, AAR88241), Sea urchin (*Strongylocentrotus purpuratus*, J. Goldstone predicted, GLEAN3_ 05931, GLEAN3_20229); Seabass (*Dicentrarchus labrax*, AAD32564); Silkworm CYP4M9 (*Bombyx mandarina*, ABK27872.1); Tunicate (*Ciona intestinalis*, JGI# Sc78); Water flea CYP4C34 (*Daphnia pulex*, BQ703383); Zebrafish CYP 4T (*Danio rerio*, Ensembl ENSDART00000013654), CYP4F13 (*Danio rerio*, Ensembl ENSDART00000063442), CYP4V2 (*Danio rerio*, Ensembl ENSDART00000089480), CYP4V7(*Danio rerio*, Ensembl ENSDART00000087976); *Cyphoma gibbosum* CYPV10v1 (EU546250), CYP4V10v2 (EU546251), CYP4BK1 (EU546252), CYP4BK2 (EU546253), CYP4BL1v1 (EU546254), CYP4BL1v2 (EU546255), CYP4BL1v3 (EU546256), CYP4BL2 (EU546264), CYP4BL3 (EU546263), CYP4BL4 (EU546257), CYP4BL5 (EU546258), CYP4BL6 (EU546259), CYP4BL7 (EU546260), CYP4BL8 (EU546261), CYP4BL9 (EU546262).
