# Supplementary material for: Cytochrome P450 diversity and induction by gorgonian allelochemicals in the marine gastropod Cyphoma gibbosum
Source: BMC Ecol. 2010 Dec 1;10:24. doi: 10.1186/1472-6785-10-24 (PMC3022543; doi:10.1186/1472-6785-10-24)
Supplement: Additional file 12 — Results of a one-way MANOVA investigating CYP4 gene expression variability among reefs for time-zero and control-fed C. gibbosum. [file 1472-6785-10-24-S12.PDF]

Additional file 11. Results of a one-way MANOVA investigating CYP4 gene expression variability among reefs for time-zero and control-fed *C. gibbosum*. Reefs were considered random factors. Statistically significant values are marked with an asterisk.

Factors	df	Wilks' Λ	*F*	p
Experiment 1 (all reefs)				
Time-zero group	20, 70	0.265	1.739	0.047*
Experiment 2 (all reefs)				
Control diet	20, 80	0.163	1.367	0.164
