# Supplementary material for: Cytochrome P450 diversity and induction by gorgonian allelochemicals in the marine gastropod Cyphoma gibbosum
Source: BMC Ecol. 2010 Dec 1;10:24. doi: 10.1186/1472-6785-10-24 (PMC3022543; doi:10.1186/1472-6785-10-24)
Supplement: Additional file 13 — Results of a two-way MANOVA investigating differences in digestive gland CYP4 gene expression in C. gibbosum feeding on control versus gorgonian diets. [file 1472-6785-10-24-S13.PDF]

Additional file 12. Results of a two-way MANOVA investigating differences in digestive gland CYP4 gene expression in *C. gibbosum* feeding on control versus gorgonian diets.

Factors	df	Wilks' Λ	*F*	p
Control vs. *B. asbestinum*				
Diet	4, 33	0.933	0.590	0.672
Reef	16, 101	0.536	1.435	0.140
Diet x Reef	16, 101	0.744	0.644	0.840
Control vs. *E. mammosa*				
Diet	4, 33	0.887	1.015	0.414
Reef	16, 98	0.598	1.127	0.341
Diet x Reef	16, 98	0.594	1.142	0.329
Control vs. *G. ventalina*				
Diet	4, 33	0.303	18.99	<0.001*
Reef	16, 101	0.116	6.476	<0.001*
Diet x Reef	16, 101	0.118	6.392	<0.001*
Control vs. *P. acerosa*				
Diet	4, 30	0.816	1.682	0.180
Reef	16, 92	0.470	1.615	0.080
Diet x Reef	16, 92	0.580	1.126	0.344
Control vs. *P. americana*				
Diet	4, 32	0.546	6.653	<0.001*
Reef	16, 98	0.574	1.227	0.261
Diet x Reef	16, 98	0.586	1.175	0.301
Control vs. *P. elisabethae*				
Diet	4, 15	0.480	4.067	0.019
Reef	8, 30	0.381	2.328	0.045
Diet x Reef	8, 30	0.619	1.017	0.444
Control vs. *P. homomalla* (all reefs included)				
Diet	5, 30	0.445	7.479	<0.001*
Reef	20, 100	0.279	2.355	<0.001*
Diet x Reef	20, 100	0.270	2.429	<0.001*
Control vs. *P. homomalla* (deletion of Shark Rock individuals)				
Diet	5, 24	0.387	7.606	<0.001*
Reef	15, 66	0.439	1.542	0.115
Diet x Reef	15, 66	0.421	1.632	0.089

Asterisks denote statistical significance following a Bonferroni correction (α = 0.002)
