# Supplementary material for: Cytochrome P450 diversity and induction by gorgonian allelochemicals in the marine gastropod Cyphoma gibbosum
Source: BMC Ecol. 2010 Dec 1;10:24. doi: 10.1186/1472-6785-10-24 (PMC3022543; doi:10.1186/1472-6785-10-24)
Supplement: Additional file 14 — Results of ANOVA comparisons (Univariate F-tests) of diet- and reef-specific mean CYP4 gene expression in C. gibbosum feeding on control vs. gorgonian diets. [file 1472-6785-10-24-S14.PDF]

Additional file 13. Results of ANOVA comparisons (Univariate F-tests) of diet- and reef-specific mean CYP4 gene expression in *C. gibbosum* feeding on control vs. gorgonian diets.

Diet comparisons	Gene(s)	df	MS	*F*	p
Control vs. *G. ventalina*					
Diet	CYP4V10	1, 36	0.016	0.050	0.825
	CYP4BK	1, 36	0.030	0.550	0.464
	CYP4BL (subA)	1, 36	6.070	46.339	<0.001*
	CYP4BL (subB)	1, 36	0.010	1.368	0.250
Reef	CYP4V10	4, 36	0.069	0.211	0.931
	CYP4BK	4, 36	0.054	0.989	0.426
	CYP4BL (subA)	4, 36	3.127	23.869	<0.001*
	CYP4BL (subB)	4, 36	0.124	1.710	0.169
Diet x Reef	CYP4V10	4, 36	0.280	0.850	0.503
	CYP4BK	4, 36	0.034	0.629	0.645
	CYP4BL (subA)	4, 36	3.555	27.137	<0.001*
	CYP4BL (subB)	4, 36	0.032	0.447	0.774
Control vs. *P. americana*					
Diet	CYP4V10	1, 35	0.037	0.114	0.738
	CYP4BK	1, 35	0.035	0.871	0.357
	CYP4BL (subA)	1, 35	0.948	6.681	0.014
	CYP4BL (subB)	1, 35	0.354	5.667	0.023
Control vs. *P. homomalla* (*All five reefs included*)					
Diet	CYP4V10	1, 34	0.162	0.442	0.511
	CYP4BK	1, 34	1.557	36.592	<0.001*
	CYP4BL (subA)	1, 34	0.394	1.316	0.259
	CYP4BL (subB)	1, 34	0.494	8.210	0.007
	CYP4BL	1, 34	1.678	19.924	<0.001*
Reef	CYP4V10	4, 34	0.643	1.756	0.161
	CYP4BK	4, 34	0.043	1.017	0.412
	CYP4BL (subA)	4, 34	3.200	10.698	<0.001*
	CYP4BL (subB)	4, 34	0.041	0.688	0.605
	CYP4BL	4, 34	0.078	0.925	0.461
Diet x Reef	CYP4V10	4, 34	0.053	0.144	0.965
	CYP4BK	4, 34	0.090	2.113	0.101
	CYP4BL (subA)	4, 34	2.354	7.870	<0.001*
	CYP4BL (subB)	4, 34	0.036	0.592	0.670
	CYP4BL	4, 34	0.144	1.713	0.170
Control vs. *P. homomalla* (*deletion of Shark Rock individuals*)					
Diet	CYP4V10	1, 28	0.097	0.488	0.490
	CYP4BK	1, 28	1.530	32.658	<0.001*
	CYP4BL (subA)	1, 28	3.877	27.577	<0.001*
	CYP4BL (subB)	1, 28	0.509	7.610	0.010
	CYP4BL	1, 28	1.765	18.754	<0.001*

Asterisks denote statistical significance following a Bonferroni correction ($\alpha = 0.007$)
