# Supplementary material for: Cytochrome P450 diversity and induction by gorgonian allelochemicals in the marine gastropod Cyphoma gibbosum
Source: BMC Ecol. 2010 Dec 1;10:24. doi: 10.1186/1472-6785-10-24 (PMC3022543; doi:10.1186/1472-6785-10-24)
Supplement: Additional file 15 — Leukotriene B4 hydroxylase activity in Cyphoma microsomes. [file 1472-6785-10-24-S15.PDF]

Additional file 14. Leukotriene B_4 hydroxylase activity in *Cyphoma* microsomes

Cyphoma individual	Diet	Activity (pmol mg^{-1} min^{-1})[a]
1	Control	nd
2	Control	nd
3	Control	nd
4	Control	0.40
5	Control	nd
6	Control	nd
7	Control	nd
8	Control	nd
9	Control	nd
10	Control	nd
11	*P. homomalla*	nd
12	*P. homomalla*	1.57
13	*P. homomalla*	0.20
14	*P. homomalla*	0.11

[a]Reporting the average activity of two technical replicates; nd (not dectected). Detection limit of the assay is 0.008 pmol mg^{-1} min^{-1}.
