# Supplementary material for: Cytochrome P450 diversity and induction by gorgonian allelochemicals in the marine gastropod Cyphoma gibbosum
Source: BMC Ecol. 2010 Dec 1;10:24. doi: 10.1186/1472-6785-10-24 (PMC3022543; doi:10.1186/1472-6785-10-24)
Supplement: Additional file 16 — Leukotriene B4 hydroxylase activity of heterologously expressed Cyphoma CYP4 proteins. [file 1472-6785-10-24-S16.PDF]

Additional file 15. Leukotriene B_4 hydroxylase activity of heterologously expressed *Cyphoma* CYP4 proteins

Gene	Clone	Induction (hrs)[a]	Activity at 23°C (pmol/mg/min)	Activity at 30°C (pmol/mg/min)	Catalytic activity (pmol/min/nmol P450)[b]
Control	--	8	nd	nd	
CYP4BK1	198_58	8	nd	nd	
CYP4BK2	198_27	8	nd	nd	
CYP4BL1	197_52	8	nd	0.0173	0.146
CYP4BL3	197_53	8	0.1180	0.1231	4.40[c]
CYP4BL4	197_48	8	nd	nd	
Control	--	15	nd	nd	
CYP4BK1	198_58	15	nd	nd	
CYP4BK2	198_27	15	nd	nd	
CYP4BL1	197_52	15	0.1357	0.2737	n/a
CYP4BL3	197_53	15	0.1827	0.2996	n/a
CYP4BL4	197_48	15	nd	nd	

Values are the average of two technical replicates; nd (not detected)
[a] Indicates time (in hours) yeast were induced on galactose media
[b] Values not applicable (n/a) due to the lack of detection of a peak at 450 nm in P450 content assays
[c] Average of values at both 23°C and 30°C
