# Supplementary material for: Cytochrome P450 diversity and induction by gorgonian allelochemicals in the marine gastropod Cyphoma gibbosum
Source: BMC Ecol. 2010 Dec 1;10:24. doi: 10.1186/1472-6785-10-24 (PMC3022543; doi:10.1186/1472-6785-10-24)
Supplement: Additional file 17 — Homology models CYP4BL3 and CYP4BK1 depicting putative substrate access channels. [file 1472-6785-10-24-S17.PDF]

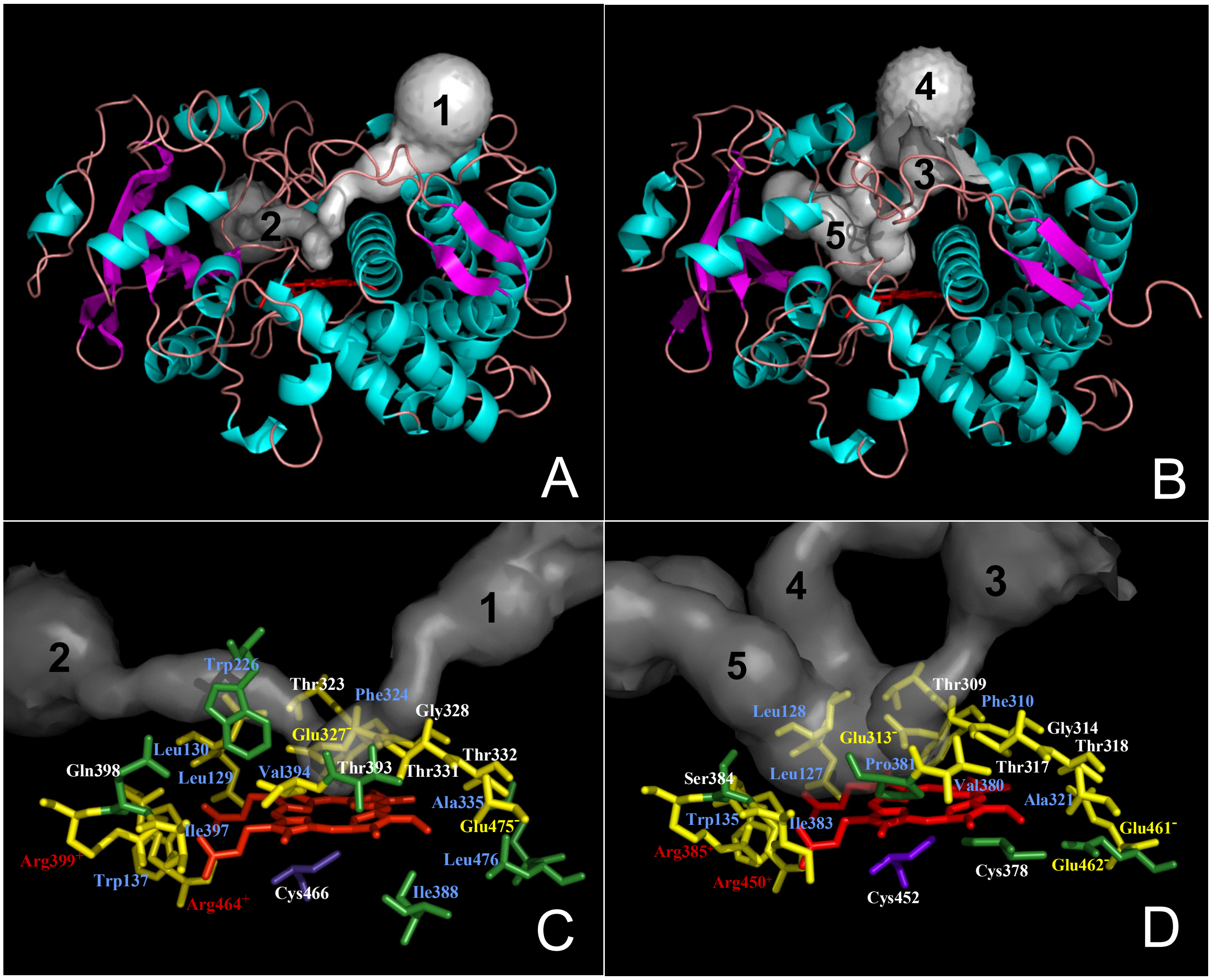

Additional file 16. Homology models CYP4BL3 and CYP4BK1 depicting putative substrate access channels. The protein backbones of CYP4BL3 (part A) and CYP4BK1 (part B) are represented by ribbon drawings with α-helices (blue), β-sheets (pink) and the Fe-heme (red) visible. Proteins are viewed along the I helix and substrate access channels are visualized in grey and numbered accordingly. A detailed view of the residues within 5 Å of the heme in CYP4BL3 (part C) and CYP4BK1 (part D) are shown in yellow with residues differing between CYP4BL3 and CYP4BK1 in green. The Fe-heme and conserved cysteine are shown in red and purple, respectively. Residues are labeled according to their biochemical properties with blue indicating hydrophobic amino acids, red indicating positively charged amino acids, yellow indicating negatively charged amino acids, and white indicating polar, uncharged amino acids.
