# Supplementary material for: Cytochrome P450 diversity and induction by gorgonian allelochemicals in the marine gastropod Cyphoma gibbosum
Source: BMC Ecol. 2010 Dec 1;10:24. doi: 10.1186/1472-6785-10-24 (PMC3022543; doi:10.1186/1472-6785-10-24)
Supplement: Additional file 18 — Deduced amino acid alignment of Cyphoma CYP4V, CYP4BK and CYP4BL consensus sequences. [file 1472-6785-10-24-S18.PDF]

Additional file 17. Deduced amino acid alignment of *Cyphoma* CYP4V, CYP4BK and CYP4BL consensus sequences. Amino acid sequences were generated from consensus nucleotide sequences and aligned using ClustalX. Grey boxes indicate areas of identical sequence among all cDNAs. Amino acid residues identical to CYP4BL9 are indicated by a "●" and gaps by a "~". The stop codon is denoted by an asterisk.

```
CYP4V10v1  1    ~~~~~~~MFV....VGIVT..GTL.WMLQ~~~~TAKFRQQISLI..PPTIPL.GNVHQMTT..GWYKQVLKWG.EFR.QGI.RIMLIS~~KPVIGVTCAE  87
CYP4V10v2  1    ~~~~~~~MFV....VGIVT..GTL.WMLQ~~~~TAKFRQQISLI..PPTIPL.GNVHQMTT..GWYKQVLKWG.EFR.QGI.RIMLIS~~KPVIGVTCAE  87
CYP4BK1    1    ..LG..SMSLT.QAV.VLVVTTL..GG.KRLLRYRS.L.AMSLL.....HS.IW.DL..LRNMTS.QR.SVFGKFT..F.K.Y.F.L...QANI..L...  100
CYP4BK2    1    ..LG..SMSLT.QAV.VLVVTTL..GG.KRLLRYRS.L.AMSLL.....HS.IW.DL.RLQNMTC.QR.SVYGKFT..F.K.Y.F.M...HANI..V...  100
CYP4BL1v1  1    ..........T.....V............~~~........................M..Y....~.K...YD..S.......I.A.........I.Y...  96
CYP4BL1v2  1    ..........T.....V............~~~........................M..Y....~.K...YD..S.......I.A.........I.Y...  96
CYP4BL1v3  1    ..........T.....V............~~~........................M..Y....~.K...YD..S.......I.A.........I.Y...  96
CYP4BL4    1    .............................~~~..........S.....................~.........I.K................IA.....  96
CYP4BL3    1    .............................~~~................................~...................................  96
CYP4BL2    1    ..........T.....V............~~~........................M..Y....~.K...YD..S.......I.A.........I.Y...  96
CYP4BL5    1    ..........T.....V............~~~................................~.........I.K................IA.....  96
CYP4BL6    1    .............................~~~..........S.....................~.........I.K................IA.....  96
CYP4BL7    1    ..........T.....V............~~~................................~...............................Y...  96
CYP4BL8    1    .............................~~~................................~...................................  96
CYP4BL9    1    MDDTFSQLKYALLLPVAGFIVYKVVLAIH~~~NFKLYAKTFDACPGETDFHWLYGNVHKFPGPN~EEGIQFQIDVMEKRPRFSRVWVGPFRGILVLIHPD  96

CYP4V10v1  88   TAEVM.N..KHIDKAQ~~~~E.GYLHA...T...TST.DK.RMR.KM...T...R..HDF.E.F..QTC..IE.L.H.VDKGQ~.NV.QDIAL.AL.IIC  182
CYP4V10v2  88   TAEVM.N..KHIDKAQ~~~~E.GYLHA...T...TST.DK.RMR.KM...T...R..HDF.E.F..QTC..IE.L.H.VDKGQ~.NV.QDIAL.AL.IIC  182
CYP4BK1    101  T.KDLF.TAD...FG~~~~~~.QFGIP...E...I.G..K........S........K...K.G.E.S.K..E.V.KYADKGESM.MYN.I.L.TL.MIM  194
CYP4BK2    101  T.KDLF.TAD...FN~~~~~~.EFGIP...E...I.G..K........S........K...K.G.E.S.K..E.V.KYADKGESI.MYN.I.L.TL.MIM  194
CYP4BL1v1  97   ..............................S..............................................................V.......  196
CYP4BL1v2  97   ..............................S..............................................................V.......  196
CYP4BL1v3  97   ..............................S..............................................................V.......  196
CYP4BL4    97   ..............NH....G.................................E..............F......A.......................  196
CYP4BL3    97   .....................................................................F......A.......................  196
CYP4BL2    97   ..............................S..............................................................V.......  196
CYP4BL5    97   ..............NH....G.................................E..............F......A.......................  196
CYP4BL6    97   ..............NH....G.........S..............................................................V.......  196
CYP4BL7    97   ..............................S..............................................................V.......  196
CYP4BL8    97   .....................................................................F......A.......................  196
CYP4BL9    97   LVRKVLKSSAPKPRSRFMKSVYDMALGWLGNGLLLANGSQWARSRRLLTPAFHFDILRPYVTVKNQAADVLLAKMKTHSEEKKPFETFYNVSICLFDVLL  196

CYP4V10v1  183  ETAMGQHV.A.TKES~~AYV.S.YKMCGFVEK.MRT..HYNKTMYD.FGP.EVHDHHLKIL.EFTIKV.KD.MSNFNAERAQRLLDD.N.KKDWVNC..S  280
CYP4V10v2  183  ETAMGQHV.A.TKES~~AYV.S.YKMCGFVEK.MRT..HYNKTMYD.FGP.EVHDHHLKIL.EFTIKV.KD.MSNFNAERAQRLLDD.N.KKDWVNC..S  280
CYP4BK1    195  R.AMS.SNDI.AK.ESH.YVVA.S..AD.LI..IRN.LAYNDFVYG..KN...FKEQ.HY..GI..QI.QE.Q.~~~~~~~~~~I..R~~~~~~~~~~~~  272
CYP4BK2    195  R.AMS.SNDI.TK.ESH.YVVA.S..ND.LI..IRN.LVYNDFVYG..KN...FKEQ.HY..GI..QI.QE.Q.~~~~~~~~~~I..R~~~~~~~~~~~~  272
CYP4BL1v1  197  .....H............Y............E..L.......................................~~~~~~~~~~................  286
CYP4BL1v2  197  .....H........~~~~~~~~~~~~~~~~~~~~~.......................................~~~~~~~~~~................  265
CYP4BL1v3  197  .....H............Y............E..L.......................................~~~~~~~~~~................  286
CYP4BL4    197  ..........................L..................Q........N........M......R...~~~~~~~~~~................  286
CYP4BL3    197  ......................................................................R...~~~~~~~~~~................  286
CYP4BL2    197  ......................................................................R...~~~~~~~~~~................  286
CYP4BL5    197  ..........................L...............................................~~~~~~~~~~................  286
CYP4BL6    197  .....H............Y............E..L.......................................~~~~~~~~~~................  286
CYP4BL7    197  .....H................................................................R...~~~~~~~~~~................  286
CYP4BL8    197  ......................................................................R...~~~~~~~~~~................  286
CYP4BL9    197  QCSFAYESNCQKTGQNDPHLQNVNELVELWAQRSMKPWLHFEWLFRLTSQGRRWYKLCDQVHAVSEDLIDKRRK~~~~~~~~~~ALEAKKAAGDTDNSED  286

CYP4V10v1  281  .TNCR.TRLA.L.M..FMS.~N.KVLSIDD..E.V...M...H...AAGMN.CT.LIGSDEKV.GK.C..L.RVFGNSDR~MP.M...KE.K.LEC....  378
CYP4V10v2  281  .TNCQ.TRLA.L.M..FMS.~N.KVLSIDD..E.V...M...H...AAGMN.CT.LIGSDEKV.GK.C..L.RVFGNSDR~MP.M...KE.K.LEC....  378
CYP4BK1    273  .G.P...YLD.L.I..T.K.DT.T.L......S.V.......H...A..I..I....CQH.DI.EK..Q.I.TV.K..D..E.E.S..PKFEFM.MV...  372
CYP4BK2    273  .G.S...YLD.L.I..T.K.DT.T.L......S.V.....A.H...A..I..I....CQH.DI.EK..Q.I.TV.K..D..E.E.S..PKFKFM.MV...  372
CYP4BL1v1  287  ....................................................................................................  386
CYP4BL1v2  266  ....................................................................................................  365
CYP4BL1v3  287  ....................................................................................................  386
CYP4BL4    287  ............................V.................A.....................................................  386
CYP4BL3    287  ..............................................A.....................................................  386
CYP4BL2    287  ..............................................A.....................................................  386
CYP4BL5    287  ....................................................................................................  386
CYP4BL6    287  ....................................................................................................  386
CYP4BL7    287  ..............................................A.....................................................  386
CYP4BL8    287  ..............................................A.....................................................  386
CYP4BL9    287  ESPGKKRLMCFVDVLLSARDEDGVGMTPLEIRNEADTFLFEGYDTTTSALSWTLYSLARWPEHQTLVQEEVDALLQGRSSDYITWDDLTQLPYTTACIKE  386

CYP4V10v1  379  .Q.LFPS..YFG.TT..EAQIS~~~~~SFRV.KDVTVIVFTSAI.RDTRWFPN.EHFD.D..LPE.SVGRH..A.I.....L......K...M.E..ILS  473
CYP4V10v2  379  .Q.LFPS..YFG.TT..EAQISKSSNCSFRV.KDVTVIVFTSAI.RDTRWFPN.EHFD.D..LPE.SVGRH..A.I.....L......K...M.E..ILS  478
CYP4BK1    373  GM.LHCP....S.VTQK.MI.E.~~~~~FS....SVCT..H.FNI...V..PD.WEFK....HP..TKDR.S.AF......P......H....E....LS  467
CYP4BK2    373  GM.LHCP....S.ISQK.MT.E.~~~~~FS....SLCS..HMFNI...V..PD.WAFK....HP..TTDR.S.AF......P......H..D.E....LS  467
CYP4BL1v1  387  .......................~~~~~........................................................................  481
CYP4BL1v2  366  .......................~~~~~........................................................................  460
CYP4BL1v3  387  ..............T........~~~~~........................................................................  481
CYP4BL4    387  .........L.Q...........~~~~~...........D..A.........................................................  481
CYP4BL3    387  .........L.Q...........~~~~~...........E............................................................  481
CYP4BL2    387  .........L.Q...........~~~~~...........E............................................................  481
CYP4BL5    387  .......................~~~~~........................................................................  481
CYP4BL6    387  .......................~~~~~...........E............................................................  481
CYP4BL7    387  .......................~~~~~........................................................................  481
CYP4BL8    387  .......................~~~~~........................................................................  481
CYP4BL9    387  AIRNYSTVPFIEREITEPLNLDG~~~~~HIIPAGTFIAIQIWCLHHNPTVWDRPHDYLPERFFGDNALNMDPFQYVPFSAGSRNCIGQNFAMNELKVMVA  481

CYP4V10v1  474  S..RN.KVKSCQSRE.L.PVGELILRPQ.G.FIELSA.*                512
CYP4V10v2  479  S..RN.KVKSCQSRE.L.PVGELILRPQ.G.FIELSA.*                517
CYP4BK1    468  .LLR.Y.FR...K~YPVV.KMT.IM...SGMRMFA...TPTV*            509
CYP4BK2    468  .LLR.Y.FR...K~.PVV.KMTIIM...SGMRMFA...TPTVYNNWEH*      515
CYP4BL1v1  482  .............~..........................*              521
CYP4BL1v2  461  .............~..........................*              500
CYP4BL1v3  482  .............~..........................LQVLVDTVGQQNH*  534
CYP4BL4    482  .............~............A.............*              521
CYP4BL3    482  .............~............A.............*              521
CYP4BL2    482  .............~............A.............*              521
CYP4BL5    482  .............~..........................*              521
CYP4BL6    482  .............~............A.............*              521
CYP4BL7    482  .............~............A.............*              521
CYP4BL8    482  .............~..........................*              521
CYP4BL9    482  RIFHRFTLALDPN~HEILRAPLATFKTEKDIKLLITPRKG*              521
```
